# Neural Substrates of Body Ownership and Agency during Voluntary Movement

**DOI:** 10.1523/JNEUROSCI.1492-22.2023

**Published:** 2023-03-29

**Authors:** Zakaryah Abdulkarim, Arvid Guterstam, Zineb Hayatou, H. Henrik Ehrsson

**Affiliations:** ^1^Department of Neuroscience, Karolinska Institutet, 171 77 Stockholm, Sweden; ^2^Department of Clinical Neuroscience, Karolinska Institutet, 171 77 Stockholm, Sweden; ^3^Université Paris-Saclay, CNRS, Institut Des Neurosciences Paris-Saclay, 91190 Gif-sur-Yvette, France

**Keywords:** fMRI, multisensory integration, rubber hand illusion, somatosensation, voluntary action

## Abstract

Body ownership and the sense of agency are two central aspects of bodily self-consciousness. While multiple neuroimaging studies have investigated the neural correlates of body ownership and agency separately, few studies have investigated the relationship between these two aspects during voluntary movement when such experiences naturally combine. By eliciting the moving rubber hand illusion with active or passive finger movements during functional magnetic resonance imaging, we isolated activations reflecting the sense of body ownership and agency, respectively, as well as their interaction, and assessed their overlap and anatomic segregation. We found that perceived hand ownership was associated with activity in premotor, posterior parietal, and cerebellar regions, whereas the sense of agency over the movements of the hand was related to activity in the dorsal premotor cortex and superior temporal cortex. Moreover, one section of the dorsal premotor cortex showed overlapping activity for ownership and agency, and somatosensory cortical activity reflected the interaction of ownership and agency with higher activity when both agency and ownership were experienced. We further found that activations previously attributed to agency in the left insular cortex and right temporoparietal junction reflected the synchrony or asynchrony of visuoproprioceptive stimuli rather than agency. Collectively, these results reveal the neural bases of agency and ownership during voluntary movement. Although the neural representations of these two experiences are largely distinct, there are interactions and functional neuroanatomical overlap during their combination, which has bearing on theories on bodily self-consciousness.

**SIGNIFICANCE STATEMENT** How does the brain generate the sense of being in control of bodily movement (agency) and the sense that body parts belong to one's body (body ownership)? Using fMRI and a bodily illusion triggered by movement, we found that agency is associated with activity in premotor cortex and temporal cortex, and body ownership with activity in premotor, posterior parietal, and cerebellar regions. The activations reflecting the two sensations were largely distinct, but there was overlap in premotor cortex and an interaction in somatosensory cortex. These findings advance our understanding of the neural bases of and interplay between agency and body ownership during voluntary movement, which has implications for the development of advanced controllable prosthetic limbs that feel like real limbs.

## Introduction

When you raise your arm, you automatically experience that it was you who caused the arm to lift and that the moving arm is your own. These two experiences blend so naturally during everyday voluntary behavior that we rarely think of them as distinct. However, in philosophy, cognitive science, and cognitive neuroscience, there is a long tradition of studying the sense of being in control of and causing bodily action through volition (i.e., the sense of agency; Jeannerod, 2003; Haggard, 2017), and the immediate perceptual experience of limbs and body parts as one's own (i.e., the sense of body ownership; Petkova and Ehrsson, 2010; Ehrsson, 2020), as distinct processes. Body ownership and agency are both considered to be fundamental aspects of self-consciousness and are critical for defining what it means to be a conscious embodied agent distinct from the environment. However, most previous studies have focused on these two experiences in isolation using different experimental paradigms, so little is known about how they combine during voluntary movement.

Body ownership is considered to depend on the integration of visual, somatosensory, and other sensory bodily signals into coherent multisensory percepts of one's own body through mechanisms of multisensory integration (Ehrsson et al., 2004; Blanke et al., 2015; Samad et al., 2015; Ehrsson, 2020), whereas agency relates to the association between voluntary action and outcome and has been linked to the match between the expected sensory consequences of movement and their sensory feedback (Frith et al., 2000) and the experience of volition during voluntary movement (Haggard, 2017). Previous functional magnetic resonance imaging (fMRI) studies have identified brain areas associated with the sense of body ownership and the sense of agency, where body ownership is associated with activity in a set of premotor-parieto-cerebellar regions (Ehrsson et al., 2004, 2005; Guterstam et al., 2013; Gentile et al., 2013; Limanowski and Blankenburg, 2016) and with agency related to activations in the right inferior parietal cortex, temporoparietal junction (TPJ), pre-supplementary motor area (SMA), insula (Farrer and Frith, 2002; Farrer et al., 2003; Schnell et al., 2007; David et al., 2008; Yomogida et al., 2010; Chambon et al., 2013), superior temporal gyrus (STG; Nahab et al., 2011; Uhlmann et al., 2020), and left primary sensorimotor cortex (Sperduti et al., 2011). However, previous agency imaging studies have focused on agency over external sensory events that occur as a consequence of bodily movement rather than agency experienced directly over one's moving limbs, and body ownership studies have not investigated movement (but see Tsakiris et al., 2010). Therefore, the precise functional neuroanatomical relationship between ownership and agency during simple voluntary movement remains unclear.

Here, we used the rubber hand illusion (RHI; Botvinick and Cohen, 1998) elicited by finger movements—the moving RHI (Kalckert and Ehrsson, 2012)—to investigate the neural bases of body ownership and agency within a single fMRI paradigm. To elicit this bodily illusion, the participants perform a repetitive finger movement with their hidden index finger while they observe a rubber hand placed in full view making the corresponding finger movements. After a few synchronous movements, the participants start to experience the moving rubber hand as their own and that they are directly controlling its movements voluntarily (Kalckert and Ehrsson, 2012, 2014). By manipulating the relative timing of the real and rubber hand finger movements (synchrony or asynchrony), the type of movement (active or passive), and the spatial–anatomic orientation of the rubber hand with respect to the real hand (congruent or incongruent), the sense of body ownership and agency can be individually manipulated (Kalckert and Ehrsson, 2012). Thus, we implemented a 2 × 2 × 2 factorial within-subjects experimental design with these three factors to identify active neuronal populations that reflect body ownership, agency, and their potential interaction. We hypothesized that ownership and agency should be associated with activity in different neural circuits, which is in line with previous studies, but also that their combination should be associated with overlapping and stronger activation in certain frontoparietal regions because of the integration of the two sensations.

## Materials and Methods

### Participants

Thirty healthy volunteers were recruited for the experiment. One of the participants canceled their participation at the last minute, and thus, 29 participants completed the experiment (15 males, 14 females; mean age, 28 ± 5 years). The number of participants recruited was based on previous similar studies on body illusions (Preston and Ehrsson, 2016) as well as another fMRI study with a similar 2 × 2 × 2 factorial design and eight conditions in a block design (Kilteni and Ehrsson, 2020). All the participants were right handed, which was assessed using the Edinburgh Handedness Inventory (Oldfield, 1971). The participants had normal or corrected-to-normal vision and had no history of neurologic or psychiatric illness. Informed consent was obtained before the experiment. The experiment was conducted according to the Declaration of Helsinki and was approved by the Swedish Ethical Review Authority.

### Moving rubber hand illusion setup

**Figure 1.**
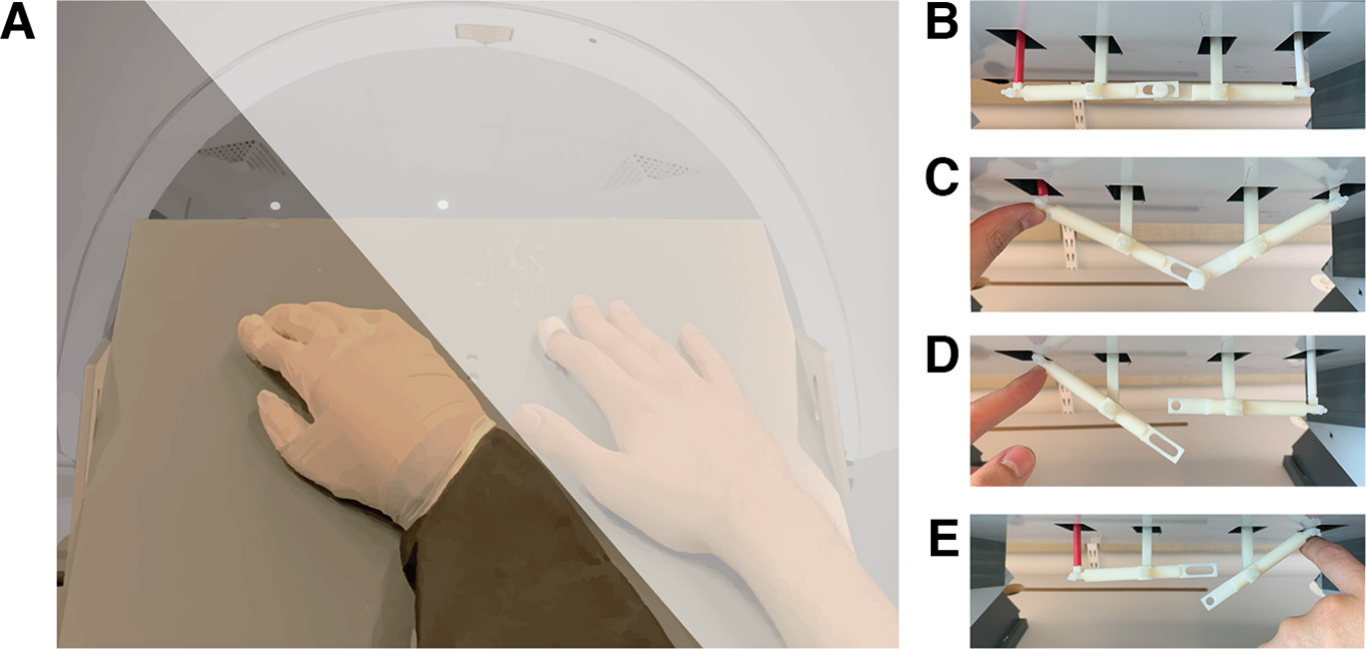
***A***, A montage of what the participants would see lying inside the MR scanner. The white semiopaque field illustrates the dark cloth that was used to cover the participant's real right hand from view. The participant's hand and the rubber hand are seen resting on a small table. The index finger of the rubber hand as well as the participant's hand are placed inside a plastic ring, which is connected to the two most lateral vertical rods seen in ***B–E***. ***B–E***, Illustration of the levers of the moving rubber hand illusion setup under the table that moved the index finger of the participant and the rubber hand. In ***B***, the levers are in a relaxed position with the index finger of the rubber hand and the participant's hand resting on the table. In ***C***, both the participant's index finger and the index finger of the rubber hand are lifted off the table. The two levers are connected to each other through a pin. In this configuration, the participants could lift their index finger, which would simultaneously lift the index finger of the rubber hand (active synchronous conditions), or the experimenter could push the index finger of the participant up by pressing on the rod underneath the participant's index finger (as seen in the image; passive synchronous condition). ***D*, *E***, The two fingers have been decoupled by removing the pin holding the two levers together. In this configuration, the index finger of the rubber hand and the participant's hand could be moved independently by the experimenter, causing delayed movements (∼0.5 s) of the index of the rubber hand in the asynchronous conditions (active and passive asynchronous conditions).

The moving rubber hand illusion setup in its original design is a vertical setup in which the participant's real hand is placed under a small table over which the rubber hand is placed (Kalckert and Ehrsson, 2012). The illusion also works in other spatial arrangements as long as the rubber hand is presented close to the real hand within peri-hand space (distance within ∼30–40 cm); we took this into consideration when redesigning the setup for the current fMRI study. The vertical setup did not fit inside the constrained space of the modern General Electric (GE) 3 T magnetic resonance (MR) scanner we used, so a horizontal version of the moving rubber hand illusion setup had to be designed. Importantly, the setup had to be able to rapidly switch between active and passive movements of the participant's index finger, as well as between synchronous and asynchronous movements of the participant's index finger and the index finger of the rubber hand. To achieve this, a new mechanical design consisting of two levers, two supports, and a plastic pin was developed (Fig. 1*A–D*). By removing the plastic pin connecting the levers, the movements of the participant's index finger could be decoupled from the movements of the index finger of the rubber hand. By having the experimenter push the lever beneath the index finger up, the fingers could be passively moved. The “rubber hand” used in our experiment was in fact a wooden hand with flexible joints (31 cm model; HAY; similar to that used in the study by Kalckert and Ehrsson, 2012). All joints of the wooden hand except the metacarpophalangeal (MCP) joint of the index finger were fixated with glue, thus only permitting movement in the MCP joint. The rubber hand was covered with a gray nitrile glove, occluding the fact that it was a wooden hand and giving it the impression of being more humanoid.

### Procedures

The participant lay comfortably in a supine position on the MR scanner bed wearing earplugs and headphones over the earplugs to protect the participant's hearing from scanner noise while allowing them to hear the instructions through headphones. The participant's head was tilted ∼30° using a custom-made wooden wedge under the head coil along with foam pads inside the head coil. The tilting of the head allowed the participants to see through the openings on the head coil and view their body from a natural (first-person) perspective. With the participant in a supine position, a small custom-made table was placed over their abdomen (fixed to the scanner bed). The participant's right hand was placed on the right side of this table, and the rubber hand was placed on the left side of this table, with the index finger of the rubber hand placed 15 cm to the left of the index finger of the participant's real hand (Fig. 1*A*). The participant's real right arm and hand were occluded by taping a dark cloth to the table and then to the roof of the MR scanner bore, thus completely hiding the participants' real hand from sight (Fig. 1*A*). The participant's right elbow was supported with a pillow to have the participants lay comfortably and not have to strain or actively maintain their arm in the required position but make it possible for them to have the arm in a relaxed posture. The rubber hand and the participant's real hand were placed parallel to each other, with the same rotations of ∼20° counterclockwise from the participants' perspective, which gave the impression of the rubber hand originating from the insertion of the participants' real arm into the torso. The participant's right index finger was placed inside a small plastic ring that was connected to a rod that in turn was connected to a lever below the table (Fig. 1). The index finger of the rubber hand was placed in an identical plastic ring and in turn connected via a separate rod to a second lever under the table. This setup allowed us to manipulate the synchrony of the movements between the rubber hand and the real hand by coupling (synchrony) or decoupling (i.e., removing the plastic pin connecting the two levers) the rubber hand from the participant's hand (Fig. 1*B–E*). This decoupling allowed the index finger of the rubber hand and the participant's hand to move independently, and thus, the experimenter could move the index finger of the rubber hand with a delay of ∼0.5 s by pressing the lever under the rubber hand up (asynchrony). Furthermore, it allowed us to manipulate whether the movement was active or passive by either having the participants lift their index finger up actively or having the experimenter push the index finger of the participant up by pressing the lever. Finally, this setup allowed us to manipulate the anatomic orientation of the rubber hand by either having the rubber hand placed in an anatomically congruent position, giving the impression of it being continuous with the body, or having the rubber hand rotated 180° to an anatomically incongruent position (Ehrsson et al., 2004).

Throughout the experiment, the participants were asked to maintain fixation on the rubber hand. The participants received verbal instructions through headphones, which consisted of two prerecorded 1-s-long audio clips of either “tap finger” or “relax.” During the active conditions, the participants were asked to perform a tapping motion with their right index finger. The tapping motion was performed by extending and then flexing the metacarpophalangeal joint while keeping the proximal and distal interphalangeal joints static; in other words, tapping with a straight finger (Kalckert and Ehrsson, 2012). In the active conditions, the tapping was self-paced, and the participants had to produce a regular rhythm of taps at ∼1 Hz without the support of a metronome or other external cues. The participants were trained to produce tapping movements at a regular speed (brief taps with short pauses between each tap) and tap gently (i.e., not forcefully press the surface). Before the scan started, the participants were trained to produce the required tapping movements in a practice trial that lasted a few minutes. In this practice trial, the participants listened to a 1 Hz metronome while tapping their right index finger in the moving rubber hand illusion condition and were then asked to continue tapping without the metronome. Self-paced tapping was used to ensure internally generated movement rather than “externally triggered” movement (Passingham, 1993), thereby avoiding potential interactions between external cue processing and agency. Participants were also trained to generate the tapping movement with a certain amplitude (3 cm; see further below). If the participants failed to maintain a reasonably consistent tapping frequency or amplitude, they received feedback from the experimenter and performed one more practice trial until the participants were consistent and reliable in their tapping frequency and amplitude throughout the trial. In the passive conditions, the participants were relaxing their index finger, and the experimenter generated the movements as described above. In these passive conditions, the experimenter matched the frequency of the participant's self-paced movements in the preceding active condition. To ensure that the amplitude of each tap that the experimenter produced was consistent, the experimenter was guided by a measuring stick taped to the table that showed the 3 cm movement amplitude target (see further below). In all conditions, the experimenter was hidden from the sight of the participant by standing on the left side of the scanner bore behind the cloth that also occluded the view of the participants' real hand (Fig. 1*A*). The experimenter received continuous instructions about the onset and end of the conditions through headphones as well as through text on a screen that displayed the next condition (the screen was placed in the control room and facing scanner through the glass window of the control room so that it could be seen from the location of the experimenter inside the scanner room).

### Movement registration and optical sensor

Underneath the index finger of the participant, ∼3 mm proximal to the hole that the rod connected to the plastic ring passed through, there was another small hole (diameter, 2 mm). In this smaller hole, a fiber optic cable attached to an optical sensor (model E3X-HD11, Omron Industrial Automation) was placed, which was able to register when the participant's index finger was lifted off the table and when it returned to the table during the tapping movements. The optical sensor registered the luminance from the fiber optic cable with preset thresholds so that it recorded dichotomic on/off data (finger on or lifted off) with a sampling frequency of 100 Hz and saved this to a text file. This allowed us to record the frequency of taps, the duration of each tap and the total number of taps in each participant and in each condition. As described above, the experimenter had a measuring stick taped to the table and could visually inspect that the participant's taps reached the same amplitude of ∼3 cm, ensuring that the amplitude of the taps was consistent across conditions.

### Design

**Figure 2.**
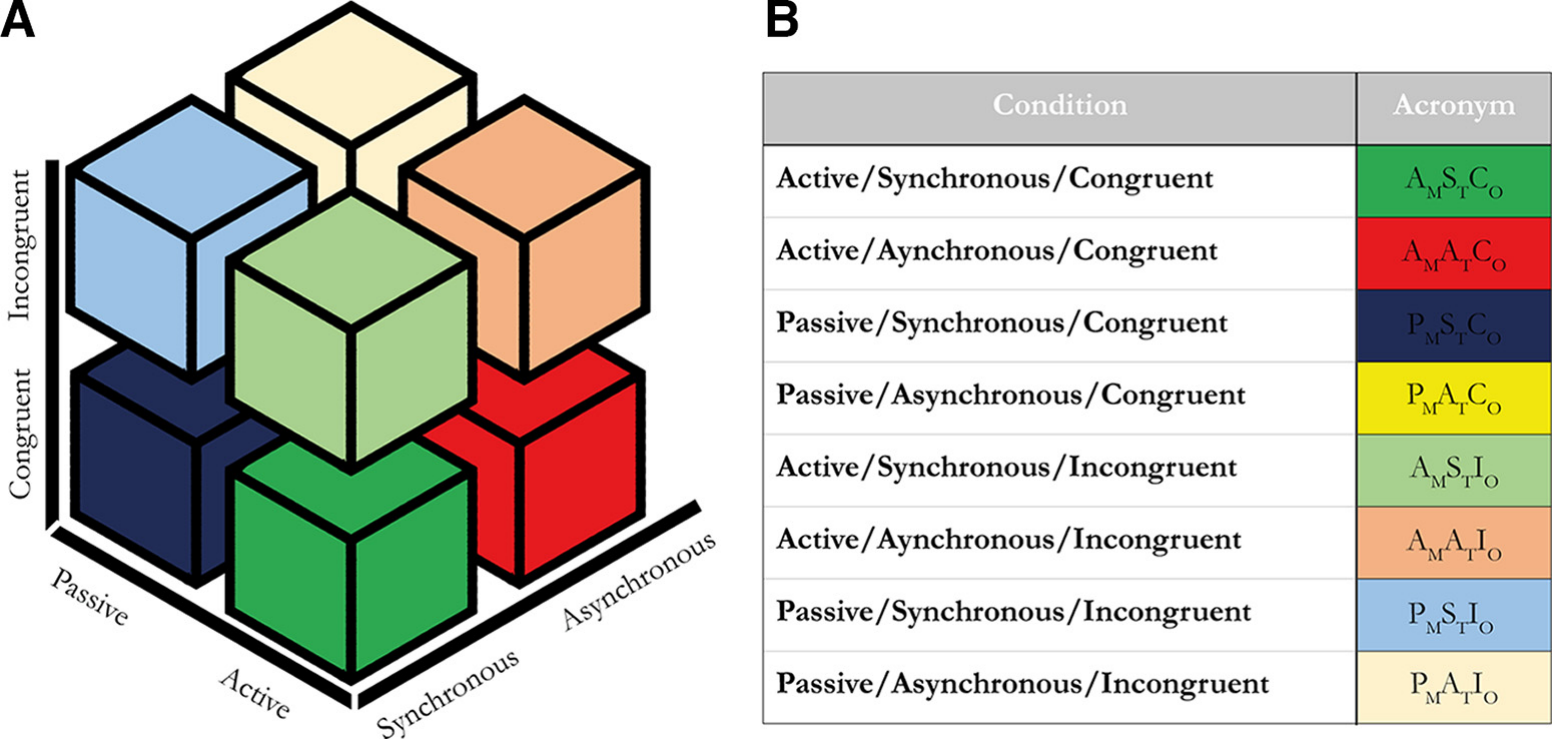
***A***, Schematic illustration of the design matrix for the 2 × 2 × 2 factorial giving rise to eight unique conditions. ***B***, All eight unique conditions and their acronyms used in this article. Each letter indicates the movement type (active or passive), the timing of the movements (synchronous or asynchronous), and the orientation of the rubber hand relative to the participant's hand (congruent or incongruent), and is followed by a subscripted letter indicating which factor the letter belongs to. M, Movement type; T, timing; O, orientation.

To test the hypothesis that the sense of agency and the sense of body ownership have different neural substrates and identify possible neural interactions when the two co-occur, we opted for a full factorial design with 2 × 2 × 2 conditions, with the factors movement type [M; active (A)/passive (P)], timing [T; synchronous (S)/asynchronous (A)], and orientation [O; congruent (C)/ incongruent (I); Fig. 2*A*], giving rise to eight unique conditions (Fig. 2*B*). The rationale behind this design is that it allows for independent manipulation of body ownership and agency by manipulating only three experimental parameters in otherwise equivalent conditions, made possible by the fact that illusory body ownership and agency in the moving rubber hand illusion follow different perceptual-cognitive rules.

Illusory body ownership depends on multisensory temporal and spatial congruence rules so that when visual and somatosensory information is matching, the brain will combine these sensory signals and illusory hand ownership is experienced, but when the incongruence between visual and somatosensory information is too great, these signals will be segregated, and illusory ownership will not be evoked (Ehrsson, 2012; Blanke et al., 2015; Samad et al., 2015; Ismail and Shimada, 2016; Fang et al., 2019; Chancel et al., 2022a). Thus, synchronously seen finger movements of the rubber hand and corresponding felt movements of the real hand elicit illusory hand ownership when the rubber hand is presented in the same spatial orientation as the participant's hidden real hand (congruent), whereas asynchrony with a 0.5 s delay (Ismail and Shimada, 2016) or presenting the rubber hand in an incongruent orientation, rotated 180° with respect to the real hand (Ehrsson et al., 2004; Kalckert and Ehrsson, 2012; Ide, 2013), breaks the body ownership illusion. Active or passive movements can both be used to trigger the moving rubber hand illusion, so the factor movement type does not determine the sense of body ownership (Kalckert and Ehrsson, 2012). Thus, illusory body ownership corresponds to the interaction of the factors of timing (T) and orientation (O) in our 2 × 2 × 2 factorial design, elicited in the two conditions with synchronous visuosomatic sensory feedback and congruent rubber hand orientation (A_M_S_T_C_O_ and P_M_S_T_C_O_; Fig. 2*B*).

Agency relates to another pair of factors in our design, namely, movement type and timing. Active movement is required for a sense of agency because active movement production is associated with volition, a prerequisite for agency (Haggard, 2017). Agency also requires a match between the expected sensory feedback from active movements and the actual sensory feedback (Frith et al., 2000). Thus, synchronously seen rubber hand movements and felt active movements of the real hand evoke a sense of agency over the rubber hand, whereas asynchrony or passive movements break the agency experience (Kalckert and Ehrsson, 2012). The orientation of the rubber hand does not matter, and one can experience agency over the finger movement of the rubber hand when the rubber hand is presented in a spatially incongruent orientation (Kalckert and Ehrsson, 2012). Thus, agency is captured by the interaction of movement type and timing in our design and will be experienced in the two conditions with active and synchronous movements (A_M_S_T_C_O_ and A_M_S_T_I_O_; Fig. 2*B*).

Thus, combined with fMRI, the 2 × 2 × 2 factorial design allowed us to isolate neural correlates of the sense of agency and the sense of body ownership (as the two-way interactions) while controlling for basic effects related to differences between active and passive movements, visuosomatosensory and visuomotor synchrony, and visual impressions from observing the rubber hand in different orientations (captured by the three main effects) and to examine possible (three-way) interaction between body ownership and agency when combined in the active moving RHI condition (A_M_S_T_C_O_). Since the two two-way interactions defining ownership and agency are orthogonal in this design, we can also examine their overlap in activation by using a conjunction analysis. Thus, we reasoned that this experimental design would be ideal for addressing the questions we were interested in.

### Behavioral experiment

Before the fMRI experiment, all subjects participated in a behavioral experiment. The rationale was fourfold: (1) we wanted to verify that the behavioral paradigm worked as expected for the purpose of the fMRI design; (2) we wanted to quantify ownership and agency using the extensive questionnaires that have been used in previous studies and that are unpractical to use during the scan sessions; (3) since the current eight conditions have never been tested in a single within-subjects design before (Kalckert and Ehrsson, 2012, tested the various conditions we use in separate experiments), we also wanted to test for a possible interaction between ownership and agency; and (4) we wanted to register how rapidly the moving RHI was induced in this group of participants exposed to the current paradigm to take this into account in the later fMRI analysis.

Thus, in this behavioral experiment, the participants were tested with the identical moving rubber hand illusion setup that would be used in the MR scanner, but lying on a bed in our behavioral testing laboratory instead. The positions of the participant's limbs, head, and body were the same as during the MR scans. The participants had all eight conditions repeated once and received a 16-statement questionnaire at the end of each condition that probed the illusory experience of the sense of body ownership and the sense of agency (Table 1; based on the study by Kalckert and Ehrsson, 2012). Control questions probing suggestibility and task compliance were also included. The questionnaire was rated on a 7-point Likert scale ranging from −3 to +3, with −3 corresponding to “completely disagree,” +3 corresponding to “completely agree,” and 0 corresponding to “neither agree nor disagree.” The stimulation period for each condition was 45 s. When all conditions had been tested, six more trials, three with the active/synchronous/congruent (A_M_S_T_C_O_) condition and three with the passive/synchronous/congruent (P_M_S_T_C_O_) condition, were conducted. In these additional trials of the A_M_S_T_C_O_ and P_M_S_T_C_O_ conditions, the illusion was induced in the same manner, but this time, the participants were instructed to verbally indicate when they started to experience that the “rubber hand was their hand” (corresponding to the fourth statement in the ownership questionnaire, Table 1; Ehrsson et al., 2004). This yielded average “illusion onset time” measurements for each participant in both the A_M_S_T_C_O_ and P_M_S_T_C_O_ conditions (Extended Data Table 3-1). These individual time intervals (A_M_S_T_C_O_: range, 0–30 s; mean, 11.5 ± 8.2 s; P_M_S_T_C_O_: range, 0–30.2 s; mean, 12.26 ± 9.1 s; nonsignificant difference between onset time in A_M_S_T_C_O_ and P_M_S_T_C_O_: *W* = 127.00; *p* = 0.346; rank–biserial correlation, −0.218) were then used to define the start of the illusion conditions of interest in the fMRI analyses (see below). This allowed us to focus our analysis on the periods when the moving rubber hand illusion had been elicited (Ehrsson et al., 2004). The periods before the illusion onset times were modeled as conditions of no interest and were not used in the statistical contrasts.

10.1523/JNEUROSCI.1492-22.2023.tab3-1 Table 3-1 Illusion onset time measurements for each participant in both the A_M_S_T_C_O_ and P_M_S_T_C_O_ conditions. Download Table 3-1, DOCX file.

**Table 1.** The statements used in the questionnaire experiment conducted before the fMRI study (the “behavioral pretest”)

	Statement
Ownership	I felt as if I was looking at my own hand
	I felt as if the rubber hand was part of my body
	It seemed as if I were sensing the movement of my finger in the location where the rubber finger moved
	I felt as if the rubber hand was my hand
Agency	The rubber hand moved just like I wanted it to, as if it was obeying my will
	I felt as if I was controlling the movements of the rubber hand
	I felt as if I was causing the movement I saw
	Whenever I moved my finger I expected the rubber finger to move in the same way
Ownership control	I felt as if my real hand were turning rubbery
	It seems as if I had more than one right hand
	It appeared as if the rubber hand were drifting toward my real hand
	It felt as if I had no longer a right hand, as if my right hand had disappeared
Agency control	I felt as if the rubber hand was controlling my will
	I felt as if the rubber hand was controlling my movements
	I could sense the movement from somewhere between my real hand and the rubber hand
	It seemed as if the rubber hand had a will of its own

Each statement was rated once per condition. The statements were rated on a 7-point Likert scale ranging from −3 to 3. There were four statements assessing the sense of body ownership and the sense of agency, as well as four control statements for both the sense of body ownership and the sense of agency.

### fMRI experiment

**Figure 3.**
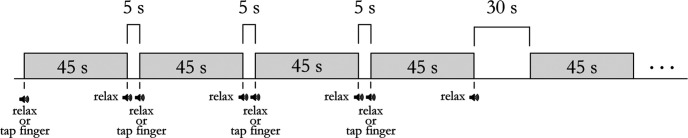
Schematic illustration of the fMRI block design. Each stimulus block consisted of one of the eight conditions with 45 s of continuous finger tapping, either actively or passively. Between each block, there was a 5 s rest baseline. After every four blocks, there was a 30 s rest condition. Four of the eight conditions were repeated four times in each run since the congruent and incongruent conditions were split into separate runs. The participants received auditory instructions at the beginning and end of each block that consisted of a 1-s-long prerecorded voice saying, “tap finger” or “relax.”

The fMRI experiment was designed as a block design, given the efficiency of this design type (Friston et al., 1999). The experiment was divided into four runs, where two runs were collected with the rubber hand in the congruent position, and two runs with the rubber hand in the incongruent position. The separation of the congruent and incongruent trials in separate runs was done because it took approximately a minute to properly reorient the rubber hand, which made it unfeasible to do within a run. The order of the runs was randomized. Each block (epoch) contained a stimulation period of 45 s followed by a 5 s resting baseline before the next condition. Each run contained four repetitions of each of the four conditions in said run, totaling eight blocks per condition per participant. Every four blocks, there was a 30 s block of a rest baseline condition in which the participants looked at the rubber hand without performing or observing any movement (Fig. 3).

### fMRI data acquisition

The experiment was conducted using a 3 tesla GE MR750 scanner equipped with an eight-channel head coil. T2*-weighted gradient echo EPIs with BOLD contrast were used as an index of brain activity (Logothetis et al., 2001; Logothetis, 2003). Each functional volume consisted of 43 continuous slices with a slice thickness of 3 mm and an interslice space of 0.5 mm. The field of view (FOV) was defined as a matrix with dimensions of 72 × 72 (in-plane resolution, 3 × 3 mm; TE, 30 ms), thus ensuring coverage of the whole brain. One volume was collected every 2.048 s (TR, 2048 ms), and a total of 1812 functional volumes were collected for each participant, divided into four runs of 453 volumes each. A high-resolution structural image was collected for each participant at the end of the experiment (3D MPRAGE sequence; 1 × 1 × 1 mm voxel size; FOV, 240 × 240 mm; 180 slices; TR, 6404 ms; TE, 2808 ms; flip angle, 12°).

### Statistical analysis

#### Questionnaire data.

The data from the behavioral pretest experiment were tested for normality using Shapiro–Wilk test. If the data deviated from normality, the results were analyzed using the nonparametric Wilcoxon signed-rank test. The questionnaire data from the pretesting were analyzed using JASP (version 0.11.1, 2019; University of Amsterdam, The Netherlands). For each participant, the subjective ratings from the four statements probing ownership were averaged into an ownership score, the four agency statements into an agency score, and the control statements were similarly averaged into an ownership control score and agency control score (Kalckert and Ehrsson, 2012). For each condition, a sense of body ownership or agency was defined as a mean ownership or agency score of >0. To test for body ownership or agency within a condition and control for unspecific effects, the ownership score was compared statistically to the ownership control score, and the agency score to the agency control score, respectively. To compare body ownership and agency between conditions, an ownership index and agency index were calculated. The indices were defined as the difference between the ownership score and the ownership control score (ownership index) and between the agency score and agency control score (agency index), respectively (Abdulkarim and Ehrsson, 2016).

#### Movement sensor data.

The data from the optical sensor were analyzed using MATLAB (statistical toolbox; version 2018b; MathWorks). The optical sensor was not available for the first 10 participants (still under development because of unexpected delay), which is why we only included data from the optical sensor from 19 participants. The number of taps from each trial was extracted for participants 11–29. The frequency of taps was calculated by dividing the number of taps by the total time of each condition. The number of taps as well as the frequency of taps was then averaged across participants for each condition. The statistical analysis focused on testing for the main effects of synchrony, active or passive movements, and congruent or incongruent rubber hand orientation in terms of the frequency of taps in line with the fMRI design.

#### fMRI data preprocessing, modeling, and statistical inference.

The fMRI data from all participants were analyzed using Statistical Parametric Mapping 12 (SPM12; Wellcome Trust Center for Neuroimaging, University College London, UK). Before the functional imaging data underwent the preprocessing steps, all functional and anatomic images were rotated back to the standard position, which they deviated from because of the forward head tilt inside the scanner coil. After that, the preprocessing steps included motion correction, slice timing correction, coregistration, and normalization [to the Montreal Neurological Institute (MNI) standard brain]. The functional images were resampled to a resolution of 2 × 2 × 2 mm, and spatial smoothing was applied using a 6 mm FWHM Gaussian kernel. The statistical analysis was performed by fitting a general linear model (GLM) to the data for each participant. The hemodynamic response function was convolved with boxcar regressors for each condition of interest. Linear contrasts were defined at the individual level and exported to the second-level random-effects analysis. Importantly, we modeled the first period in each condition as a condition of no interest, based on the time it took for each individual participant to experience the illusion in the behavioral pretest (see above), and the periods from illusion onset to the end of each condition as the condition of interest used in our main analyses (in line with studies by Ehrsson et al., 2004; Guterstam et al., 2013). For the A_M_S_T_C_O_ and P_M_S_T_C_O_ conditions, we used their corresponding rubber hand illusion onset times, whereas for all other conditions (that did not trigger the rubber hand illusion), we used the average of the A_M_S_T_C_O_ and P_M_S_T_C_O_ times.

For the main contrasts, we had anatomic hypotheses regarding which regions we expected to be activated during experiences of body ownership and agency based on the previous fMRI literature (see Introduction); therefore, in these regions, we report the results that are statistically significant at a threshold of *p* < 0.05 after small-volume correction [familywise error (FWE) correction]. However, since earlier ownership studies used brushstrokes or similar tactile stimulation applied to relaxed hands instead of finger movements, we anticipated that the exact location of peaks associated with the rubber hand illusion could change within the hypothesized frontal, parietal, and subcortical regions. Therefore, the volumes of interest used in the small-volume correction were centered on peak coordinates obtained from a “localizer” study where we used the same 3 T MR scanner and fMRI scanning protocol as in the main experiment to identify the locations of active candidate areas during the moving rubber hand illusion. In brief, the localizer study included 27 participants looking at and controlling the index finger movement of a robotic hand wearing a plastic glove identical in shape and size to the rubber hand used in our current experiment. This robotic rubber hand was placed in view of the participant on a supporting table in an arrangement that was very similar to the one used in the current study. When the participant moved his or her index finger, the rubber hand moved its index finger in the same way and synchronously, triggering the moving rubber hand illusion (verified with illusion questionnaire ratings that were affirmative in most participants; data not shown). In the localizer study, we contrasted this illusion condition (corresponding to the A_M_S_T_C_O_ condition in the present study) to a resting baseline condition where the participants were just looking at the rubber hand without performing or observing any movement. Peaks from this localizer contrast were then used to define the coordinates in MNI space for the spheres (radius, 10 mm) in the small-volume corrections (Extended Data Table 12-2, list of all peaks used from this localizer study to define the volumes of interest). For the intraparietal cortex—a region often associated with the RHI and illusory hand ownership in the previous fMRI literature—we added peaks from the study by Ehrsson et al. (2004) since no activations were detected in the localizer contrast in this region. Similarly, for the left insular cortex and right angular gyrus in the TPJ region—two areas often associated with different aspects of agency in the previous literature—we used coordinates from classic neuroimaging agency studies (Farrer and Frith, 2002; Farrer et al., 2003) to define peaks for small-volume correction in these regions. In the rest of the brain (i.e., outside the regions related to our a priori defined anatomic hypotheses), we corrected for the number of comparisons in the whole brain space using a test of false discovery rate (FDR) set at *p* < 0.05. All our statistical inferences and main findings are based on results that survive multiple-comparison correction based on these two approaches, which collectively balances type 1 and type 2 errors and hypothesis-driven and explorative approaches.

Some activations that did not survive correction for multiple comparisons are still mentioned in the text or shown in figures as part of the statistical parametric maps produced by SPM12 (based on a threshold of *p* < 0.005, uncorrected). We report these for purely descriptive purposes (Gentile et al., 2013; Preston and Ehrsson, 2016) and always clearly identify these as not reaching our significance criterion. We report these nonsignificant activations mainly for the following five reasons: (1) false-negatives and limited sensitivity is a concern in fMRI studies, so being overly conservative might conceal potentially interesting results; (2) we want to report the activation maps in a transparent fashion and not only describe those regions that were part of our hypothesis; (3) activation peaks that did not survive correction for multiple comparison can still be used to define anatomic hypotheses for future fMRI studies; (4) the reporting of the entire activation maps, including nonsignificant activation, can provide information about the anatomic specificity of these latter effects (i.e., single active brain area or widespread effects in many regions); and (5) nonsignificant peaks can be used in future imaging meta-analyses where it is often important to have data from the whole brain (and not only a few peaks that survive multiple-comparisons correction). As mentioned, all main conclusions in the article are based on activations that are significant (in one case, almost significant) after correction for multiple comparisons (i.e., *p* < 0.05 after FWE correction).

The visualization of the results is performed by overlaying the peaks on a 3D rendering of a standard MNI brain using Surf Ice (https://www.nitrc.org/projects/surfice/) as well as on sections from the average anatomic image for all participants. The anatomic localizations of the activations were based on macroanatomical landmarks (sulci and gyri) using the terminology from Duvernoy's brain atlas (Duvernoy, 1999). For peaks in the cerebellum, we used the SUIT toolbox for anatomic localization based on a probabilistic atlas of the cerebellum (Diedrichsen et al., 2009, 2011). All coordinates for the activation peaks are given in MNI space. Contrast estimates for each significant peak were extracted using MATLAB (version 2018b) and are presented in bar charts together with the corresponding SEs for purely descriptive purposes. In line with the SPM approach, we make no further statistical analyses on these bar chart plots, but all conclusions and statistical inferences are based on significant (two-way and three-way) interaction contrasts in line with our factorial design.

#### Planned fMRI analyses.

To identify regions that display BOLD responses that reflect the sense of body ownership or the sense of agency, we defined linear contrasts that corresponded to the two-way interactions that captured ownership and agency in our factorial design (ownership: interaction timing × orientation; agency: interaction timing × movement type). In other words, we subtracted the control conditions, where no illusory experience in question was present (or strongly suppressed), from the experimental condition in which they were present. Thus, for the sense of body ownership, we defined the contrast as [(P_M_S_T_C_O_ – P_M_A_T_C_O_) – (P_M_S_T_I_O_ – P_M_A_T_I_O_)] + [(A_M_S_T_C_O_ – A_M_A_T_C_O_) – (A_M_S_T_I_O_ – A_M_A_T_I_O_)], including both the active and passive conditions. This contrast corresponds to the interaction between the factors synchrony (of the movements) and congruency (between the orientation of the rubber hand with the participant's real hand) since we know asynchronous movements and anatomic incongruency disrupt the sense of ownership of the rubber hand in the moving rubber hand illusion (Kalckert and Ehrsson, 2012). Similarly, for the sense of agency, we defined the contrast [(A_M_S_T_C_O_ – P_M_S_T_C_O_) – (A_M_A_T_C_O_ – P_M_A_T_C_O_)] + [(A_M_S_T_I_O_ – P_M_S_T_I_O_) – (A_M_A_T_I_O_ – P_M_A_T_I_O_)], including both the congruent and incongruent conditions. This contrast is the interaction between the two factors of timing (synchronous or asynchronous) and type of movement (active or passive) because we know that both the sense of volition associated with active movements and the match between expected and actual sensory feedback from the movements are required for a sense of agency to develop (Haggard, 2017); hence, both asynchronous movements and passive movements should abolish the sense of agency of the rubber hand. Note that these key contrasts are balanced and fully matched in terms of the magnitude of visual and somatosensory stimulation related to the observed and felt movements, as well as the frequency and amplitude of finger taps (Extended Data Table 13-1), and thus isolate the neural activities related to ownership and agency in which we are interested.

10.1523/JNEUROSCI.1492-22.2023.tab13-1 Table 13-1 Frequency of taps per condition and pre-illusion period. Download Table 13-1, DOCX file.

A further strength of this design is that the two interaction contrasts that operationalize ownership and agency are orthogonal (i.e., independent), which means that we can also test for active voxels that are significantly active in both contrasts by using a conjunction analysis. Thus, this conjunction analysis identifies active areas that show increases in activity that reflect both ownership and agency. To investigate this, we conducted a conjunction by performing a one-way ANOVA at the second-level analysis and entering the two different first-level contrasts as the groups in the oneway ANOVA. The contrasts are then specified for the two groups ([0, 1] and [1, 0]) and both contrasts are selected and displayed as a conjunction at the second level.

Finally, the current 2 × 2 × 2 factorial design allows us to investigate the interaction between the sense of body ownership and the sense of agency. To this end, we defined a linear contrast that was composed of a three-way interaction among the three factors in the factorial design, namely, movement type, timing and orientation. This contrast [(A_M_S_T_C_O_ – P_M_S_T_C_O_) – (A_M_A_T_C_O_ – P_M_A_T_C_O_)] – [(A_M_S_T_I_O_ – P_M_S_T_I_O_) – (A_M_A_T_I_O_ – P_M_A_T_I_O_)] identifies a neural response that specifically reflects the combination of body ownership and agency when voluntary moving one's body. This can reflect a stronger sense of ownership during active movements or differences in agency over one's own body part (the rubber hand during the rubber hand illusion) compared with agency over an external object (the rubber hand in the incongruent orientation that does not feel like part of one's body).

#### Post hoc fMRI connectivity analyses.

Task-related connectivity was assessed by performing a psychophysiological interaction (PPI) analysis. The PPI indices task or contrast specific changes in the connectivity between two brain regions. A significant PPI indicates that the correlation of the brain activity in the two regions (measured as the change in the slope of their linear regression curve) changes significantly with the experimental or psychological context (Friston et al., 1997). To follow-up on the regional results (see below), we decided to conduct a *post hoc* PPI analysis for purely descriptive purposes. We placed a seed voxel in the postcentral gyrus contralateral to the stimulated hand. The seed was selected based on activity in this region that was elucidated during the three-way interaction contrast described above. The seed was defined for each participant as a 10 mm sphere around the group-level activation in the postcentral gyrus. From this, the time series of activity (first eigenvariate) was extracted and entered into the PPI analysis with the contrast weights from the three-way interaction. The PPI regressors created at the individual level were analyzed at the group level using one-sample *t* tests.

#### Post hoc descriptive correlation analysis of ownership contrast and questionnaire ratings.

In a *post hoc* complementary approach, we explore a possible relationship between the subjective ratings of ownership, as rated by the individual participants in the questionnaires in the behavioral experiment (before the fMRI), and the contrast that describes the ownership-related activation. Unlike agency, which can be experienced by everybody, the feeling of ownership in the rubber hand illusion is vividly experienced in ∼60–80% of participants (Ehrsson et al., 2005; Lloyd, 2007; Kalckert and Ehrsson, 2014), making it possible to probe how individual differences in illusion strength relate to brain activation. Previous studies have shown such a relationship in the premotor cortex (Ehrsson et al., 2004, 2005; Gentile et al., 2013). To this end, we conducted analyses of the fMRI data combined with a behavioral covariate. For each participant, we calculated “behavioral contrast” (analogous to the defined contrasts in the fMRI analyses; [(P_M_S_T_C_O_ – P_M_A_T_C_O_) – (P_M_S_T_I_O_ – P_M_A_T_I_O_)] + [(A_M_S_T_C_O_ – A_M_A_T_C_O_) – (A_M_S_T_I_O_ – A_M_A_T_I_O_)]) of the ownership ratings from all eight conditions and entered this as a covariate in the GLM in the second-level analysis together with the contrast images reflecting the ownership contrast. This analysis allowed us to examine whether stronger subjective ownership in the synchronous and congruent conditions (A_M_S_T_C_O_ and P_M_S_T_C_O_ compared with the other conditions) correlated with stronger BOLD signals specifically in the ownership contrast.

## Results

### Behavioral experiment

**Figure 4.**
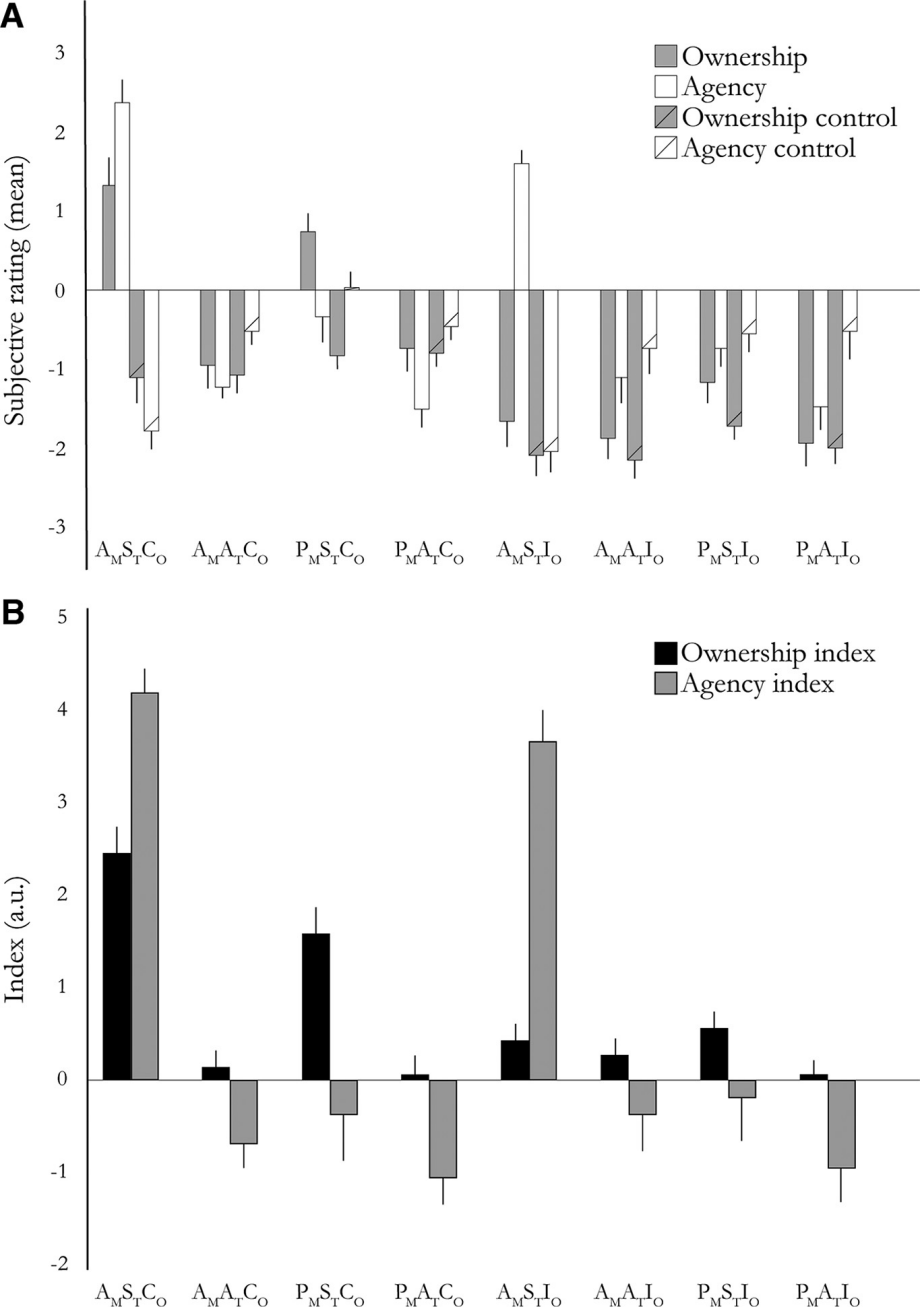
***A***, The results from the behavioral experiment. These results show a double dissociation between the sense of body ownership and sense of agency in our full factorial design. The A_M_S_T_C_O_ condition displayed high ratings for both sense of body ownership and sense of agency. The P_M_S_T_C_O_ condition showed high ownership ratings and low agency ratings, whereas the A_M_S_T_I_O_ condition showed high agency ratings and low ownership ratings. Bars represent mean ratings, and error bars indicate the SEM. ***B***, Ownership and agency indices calculated by subtracting the pooled ownership and agency control ratings from the pooled ownership and agency ratings, respectively. Bars indicate the means, and error bars indicate the SEM.

The results from the behavioral pretest experiment replicated the main findings from the original article on the moving rubber hand illusion and confirmed that our behavioral paradigm worked as expected (Kalckert and Ehrsson, 2012), but in a full 2 × 2 × 2 factorial within-subject design. The results confirmed that the sense of body ownership and sense of agency can be dissociated behaviorally, as we had expected (Fig. 4*A*). In the A_M_S_T_C_O_ condition, the participants experienced both a sense of body ownership and agency of the rubber hand (i.e., the mean rating scores of these two sensations were both positive, meaning that, on average, the participants affirmed both these experiences in the moving RHI condition with active finger movements). Furthermore, in the P_M_S_T_C_O_ condition, the moving RHI condition with passive finger movements, the participants experienced a sense of body ownership (positive rating score) of the rubber hand but denied experiencing a sense of agency (negative mean agency score). Finally, in the A_M_S_T_I_O_ condition, the participants experience a sense of agency over the rubber hand but no sense of body ownership (positive agency and negative ownership scores). In the control conditions, the participants did not report sensing body ownership or agency, and the mean ownership and agency scores were negative (Fig. 4*A*).

We then compared the ownership to the ownership control ratings and found significantly higher ratings of the ownership statements compared with the control statements in the A_M_S_T_C_O_ condition (*W* = 349; *p* < 0.001; rank–biserial correlation, 0.989) and the P_M_S_T_C_O_ condition (*W* = 314; *p* < 0.001; rank–biserial correlation, 0.932). The same analysis for the sense of agency showed significantly higher ratings of the agency statements compared with the agency control statements in the A_M_S_T_C_O_ condition (*W* = 351; *p* < 0.001; rank–biserial correlation, 1.00) and the A_M_S_T_I_O_ condition (*W* = 351; *p* < 0.001; rank–biserial correlation, 1.00). The individual ratings for each statement and condition are given in Extended Data Table 4-1.

10.1523/JNEUROSCI.1492-22.2023.tab4-1 Table 4-1 Mean ratings and standard deviation of each statement from the behavioral experiment. Download Table 4-1, DOCX file.

We then directly tested the hypothesis that the sense of body ownership depended on synchronous visuosomatosensory feedback when moving the finger as well as spatial congruency between the orientations of the rubber hand and the participants' real hand (Botvinick and Cohen, 1998; Tsakiris et al., 2010; Ehrsson, 2012; Kalckert and Ehrsson, 2012). To this end, we analyzed the ownership indices [the difference between the ownership score and ownership control score] in a 2 × 2 × 2 ANOVA (Fig. 4*B*, ownership and agency indices across the eight conditions). The factors movement type (active/passive), timing of movements (synchronous/asynchronous), and orientation of the rubber hand (congruent/incongruent) were entered into the analysis. The results showed a significant main effect of movement (*F* = 6.63; df = 29, 1; *p* = 0.016; η^2^ = 0.012), a significant main effect of timing (*F* = 41.276; df = 29, 1; *p* < 0.001; η^2^ = 0.216), and a significant main effect of orientation (*F* = 17.645; df = 29, 1; *p* < 0.001; η^2^ = 0.091). Importantly, the interaction between timing and orientation was significant (*F* = 31.933; df = 29, 1; *p* < 0.001; η^2^ = 0.109), in line with the spatial and temporal multisensory rules of illusory rubber hand ownership (Kalckert and Ehrsson, 2012) and our operationalization of ownership in the fMRI factorial experimental design. There was no significant interaction between timing and movement type (*F* = 0.894; df = 29, 1; *p* = 0.353; η^2^ = 0.002). However, the interaction between movement type and orientation was also significant (*F* = 5.982; df = 29, 1; *p* = 0.022; η^2^ = 0.008), which suggests higher ownership ratings during the active finger movements when the rubber hand was in a spatially congruent orientation. Moreover, there was a significant three-way interaction among timing, movement type, and orientation (*F* = 6.421; df = 29, 1; *p* = 0.018; η^2^ = 0.013). This three-way interaction suggests enhanced ownership of the rubber hand in the active synchronous congruent condition when participants experience both ownership and agency over the moving rubber hand compared with the passive synchronous congruent condition, when people only experience illusory ownership, and thus provides behavioral support for examining the interaction of ownership and agency in our factorial fMRI design. In line with this, *post hoc* pairwise comparisons between the A_M_S_T_C_O_ and P_M_S_T_C_O_ conditions in terms of ownership index (*t* = 3.155; df = 29; *p* = 0.004; Cohen's *d* = 0.607) and ownership scores (*t* = 2.413; df = 29; *p* = 0.023; Cohen's *d* = 0.464) further revealed significant differences in both cases. This is an interesting behavioral finding that suggests that active finger movements provide a stronger cue for body ownership than passive finger movements, which has a bearing on an ongoing debate in the behavioral literature on whether body ownership and agency interact in the moving rubber hand illusion or if they are completely independent (Dummer et al., 2009; Tsakiris et al., 2010; Walsh et al., 2011; Kalckert and Ehrsson, 2012, 2014, 2017; Riemer et al., 2013; Hara et al., 2022).

We hypothesized that the sense of agency is dependent on synchronous visuomotor feedback (i.e., the match between predicted sensory consequences of the active movement and sensory feedback) as well as on participants actively moving the index finger (i.e., voluntarily generating the movements; Kalckert and Ehrsson, 2012, 2014). To this end, we analyzed the agency indices (i.e., the difference between the agency scores and the agency control scores) in a 2 × 2 × 2 ANOVA. The three factors of movement type (active/passive), timing (synchronous/asynchronous), and orientation (congruent/incongruent) were entered in the analysis (Fig. 4*B*). As expected, the results showed a significant main effect of movement type (*F* = 42.244; df = 29, 1; *p* < 0.001; η^2^ = 0.207) and a significant main effect of timing (*F* = 107.572; df = 29, 1; *p* < 0.001; η^2^ = 0.255), which suggests that both active movements and synchronous seen and felt movement enhanced agency ratings. There was no main effect of orientation (*F* = 0.021; df = 29, 1; *p* = 0.886; η^2^ = 0.00002), indicating that the orientation of the rubber hand did not influence agency. Importantly, the interaction between synchrony and movement type was significant (*F* = 36.751; df = 29, 1; *p* < 0.001, η^2^ = 0.132), in line with the hypothesis and our operationalization of agency as this two-way interaction in our fMRI design. The interaction between movement type and orientation was not significant (*F* = 0.406; df = 29, 1; *p* = 0.530, η^2^ = 0.0005), nor was the interaction between synchrony and orientation (*F* = 0.379; df = 29, 1; *p* = 0.251, η^2^ = 0.001). The three-way interaction among synchrony, movement type, and orientation was also nonsignificant (*F* = 1.560; df = 29, 1; *p* = 0.223, η^2^ = 0.002). These latter results are consistent with the hypothesis that agency does not depend on the orientation of the rubber hand and that agency can be operationalized as an interaction between movement type and temporal congruence, only arising for active movements with synchronous visual feedback. Overall, the questionnaire results from our behavioral experiment confirmed that our selective manipulation of ownership and agency in the moving rubber hand illusion worked as expected, and was in line with established multisensory and cognitive constraints and provided behavioral support for examining the interaction of ownership and agency in the fMRI data (see below).

### The sense of body ownership is associated with activity in multisensory frontal and parietal regions as well as in cerebellar regions

**Figure 5.**
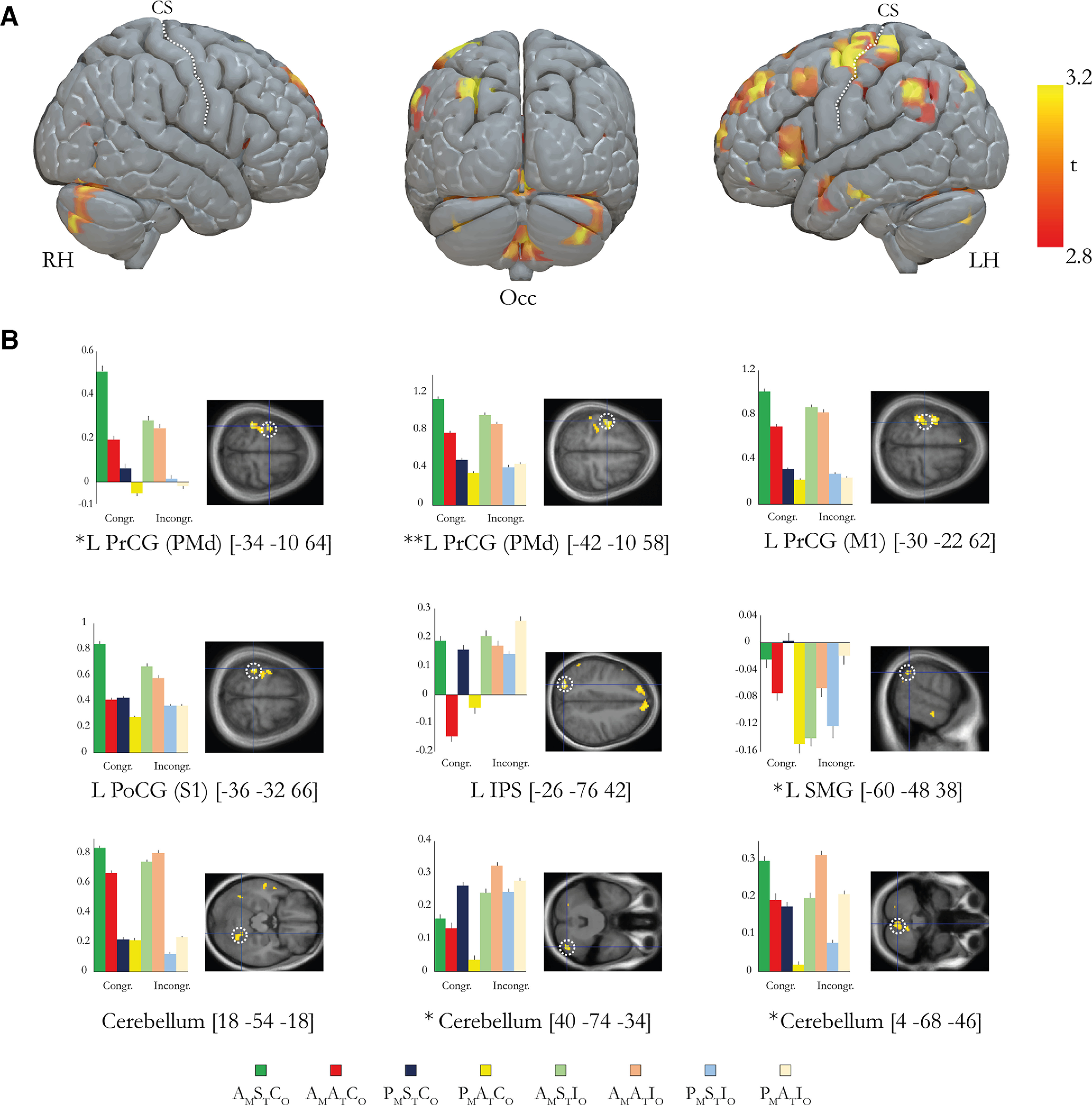
***A***, Overview of the brain regions that display activation reflecting the sense of body ownership over the rubber hand defined by the contrast [(P_M_S_T_C_O_ – P_M_A_T_C_O_) – (P_M_S_T_I_O_ – P_M_A_T_I_O_)] + [(A_M_S_T_C_O_ – A_M_A_T_C_O_) – (A_M_S_T_I_O_ – A_M_A_T_I_O_)]. For display purposes only, the activations are projected onto a three-dimensional rendering of a standard brain with a threshold of *p* < 0.005 (uncorrected for multiple comparisons, *k* ≥ 5). RH, Right hemisphere; LH, left hemisphere; Occ, occipital view; CS, central sulcus. ***B***, Bar charts displaying the parameter estimates (a.u.) and SEs for the major peaks of activation. The coordinates are given in MNI space. The peaks are displayed in representative sections indicated by a dotted white circle on an activation map (*p* < 0.005, uncorrected for display purposes). L, Left; R, right; PrCG, precentral gyrus; PoCG, postcentral gyrus; IPS, intraparietal sulcus; SMG, supramarginal gyrus. Asterisks indicate activation peaks that survive small-volume correction (*p < 0.05, corrected; **p < 0.01); the peaks without an asterisk did not survive correction and are reported in Table 2 with their uncorrected *p* value. All peaks from the contrast are reported in Extended Data Table 5-1. Condition key: first letter A or P (active or passive) with subscript M (movement); second letter S or A (synchronous or asynchronous) with subscript T (timing); and third letter C or I (congruent or incongruent) with subscript O (orientation).

To identify activations associated with the sense of ownership of the rubber hand in both the active and passive conditions, we used the contrast [(P_M_S_T_C_O_ – P_M_A_T_C_O_) – (P_M_S_T_I_O_ – P_M_A_T_I_O_)] + [(A_M_S_T_C_O_ – A_M_A_T_C_O_) – (A_M_S_T_I_O_ – A_M_A_T_I_O_)]. In line with our hypothesis, this contrast revealed significant activation peaks in the left premotor cortex, posterior parietal cortex, and cerebellum (*p* < 0.05, FWE corrected for multiple comparisons; Fig. 5, Table 2). The premotor activations were located in the precentral gyrus at a location that corresponds to the dorsal premotor cortex (PMd; −34, −10, 64; *p* < 0.05, FWE corrected; Fig. 5), and parietal lobe activations were observed in the supramarginal gyrus (SMG; −60, −48, 38; *p* < 0.05, FWE corrected; Fig. 5). Activation peaks were also observed in the primary motor cortex (precentral gyrus) and the primary somatosensory cortex (postcentral gyrus) at sites that corresponded very well to peaks identified in the localizer experiment (see above). However, since no a priori hypotheses existed for these regions and they did not survive correction for multiple comparisons at the whole-brain level, they are reported with their uncorrected *p* values. We also observed activity in the intraparietal cortex (*p* < 0.001, uncorrected) but more posteriorly than we had predicted based on previous work. In the subcortical structures, we observed significant activity in the Crus I (lobule VIIa; 40, −74, −34) and vermis (lobule VIIa; 4, −68, −46) of the cerebellum (*p* < 0.05, FWE corrected; Fig. 5). Finally, we observed a large active cluster in the left dorsolateral prefrontal cortex (Fig. 5*A*; *p* < 0.001 uncorrected). No clusters survived correction for multiple comparisons at the whole-brain level (FDR corrected). Further statistical details on the anatomic locations in MNI space of the above-mentioned peaks are shown in Figure 5 and Table 2.

**Table 2.** Activation peaks for the main contrasts

	Anatomical region	MNI *x*, *y*, *z* (mm)	Peak *t*	*p*-value
**A**				
	L PrCG (PMd)	−42, −10, 58	4.66	0.009**
	L PrCG (PMd)	−34, −10, 64	4.30	0.019*
	L PrCG (PMd)	−42, −12, 56	4.13	0.010*
	L PrCG (PMd)	−36, −10, 62	3.99	0.014*
	L SMG	−60, −48, 38	3.69	0.025*
	R Cerebellum (Vermis VIIa)	4, −68, −46	3.47	0.038*
	R Cerebellum (Crus I)	40, −74, −34	3.28	0.027*
	R Cerebellum (Crus I)	38, −72, −24	3.19	0.034*
	R ITG	42, −70, −8	3.38	0.046*
	L dlPFC	−24, 42, 38	4.42	0.001
	L mPFC	6, 50, 40	3.33	0.001
	L PrCG (M1)	−30, −22, 62	3.21	0.001
	L PoCG (S1)	−36, −22, 50	3.72	<0.001
	L PoCG	−46, −14, 52	3.51	<0.001
	L PoCG	−36, −32, 66	5.01	<0.001
	L PoCG	−56, −16, 40	3.05	0.003
	L IPS	−26, −76, 42	4.91	<0.001
	R IOG	44, −70, −12	2.97	0.003
	R Cerebellum (IV-V)	18, −54, −18	3.01	0.001
**B**				
	R STG	58, −24, 12	4.03	0.051^+^
	L PrCG (PMd)	−38, −8, 62	3.89	0.013*
	R STG	60, −20, 12	5.12	<0.001
	L STG	−50, −28, 6	4.88	<0.001
	L PoCG (S1)	−52, −30, 54	3.51	0.001
	R IPS	36, −40, 52	3.47	0.001
	L IPS	−36, −40, 46	3.69	<0.001
**C**				
	L PoCG (S1)	−38, −28, 52	4.21	0.007*
	L PoCG (S1)	−36, −26, 38	2.85	0.004
	L PoCG	−54, −18, 28	2.96	0.003
	L PoCG	−52, −16, 12	3.26	0.001
**D**				
	L MOG	−20, −94, 0	4.08	<0.001
	R MOG	26, −92, 4	3.08	0.002

**A**, The sense of body ownership in the moving rubber hand illusion is expressed as the interaction between synchrony and orientational congruency between the participant's real hand and the rubber hand, and is defined as the contrast [(P_M_S_T_C_O_ – P_M_A_T_C_O_) – (P_M_S_T_I_O_ – P_M_A_T_I_O_)] + [(A_M_S_T_C_O_ – A_M_A_T_C_O_) – (A_M_S_T_I_O_ – A_M_A_T_I_O_)]. **B**, The sense of agency is expressed as the interaction between synchrony and movement type (active/passive), and is defined as the contrast [(A_M_S_T_C_O_ – P_M_S_T_C_O_) – (A_M_A_T_C_O_ – P_M_A_T_C_O_)] + [(A_M_S_T_I_O_ – P_M_S_T_I_O_) – (A_M_A_T_I_O_ – P_M_A_T_I_O_)]. **C**, The three-way interaction among synchrony, movement type (active/passive), and orientation, representing the areas that demonstrate increased activity when experiencing agency over bodily objects as opposed to external objects, and is defined as the contrast [(A_M_S_T_C_O_ – P_M_S_T_C_O_) – (A_M_A_T_C_O_ – P_M_A_T_C_O_)] – [(A_M_S_T_I_O_ – P_M_S_T_I_O_) – (A_M_A_T_I_O_ – P_M_A_T_I_O_)]. **D**, The inverse of the three-way interaction among synchrony, movement type (active/passive), and orientation, representing the areas that demonstrate increased activity when experiencing agency over external objects as opposed to bodily objects, and is defined as the contrast [(A_M_S_T_C_O_ – P_M_S_T_C_O_) – (A_M_A_T_C_O_ – P_M_A_T_C_O_)] – [(A_M_S_T_I_O_ – P_M_S_T_I_O_) – (A_M_A_T_I_O_ – P_M_A_T_I_O_)]. PrCG, Precentral gyrus; PoCG, postcentral gyrus; ITG, inferior temporal gyrus; dlFPC, dorsolateral prefrontal cortex; mPFC, medial prefrontal cortex; IPS, intraparietal sulcus; IOG, inferior occipital gyrus; MOG, middle occipital gyrus.

*Activation peaks that survive small volume correction (FWE correction, *p* < 0.05 corrected).

**Activation peaks that survive small volume correction (FWE correction, *p* < 0.01 corrected).

^+^An activation peak in the agency contrast that almost reached statistical significance after small volume correction (FWE correction); the peaks without an asterisk did not survive correction and are reported with their uncorrected *p* value.

10.1523/JNEUROSCI.1492-22.2023.tab5-1 Table 5-1 All peaks from the ownership contrast *[(P_M_S_T_C_O_-P_M_A_T_C_O_)-(P_M_S_T_I_O_-P_M_A_T_I_O_)] + [(A_M_S_T_C_O_-A_M_A_T_C_O_)-(A_M_S_T_I_O_-A_M_A_T_I_O_)]*. All peaks that survived the threshold p<0.005 (uncorrected, k≥10) are reported. Three peaks located in the white matter were excluded from this table. Download Table 5-1, DOCX file.

### Correlation between subjective ownership ratings and ownership contrast

**Figure 6.**
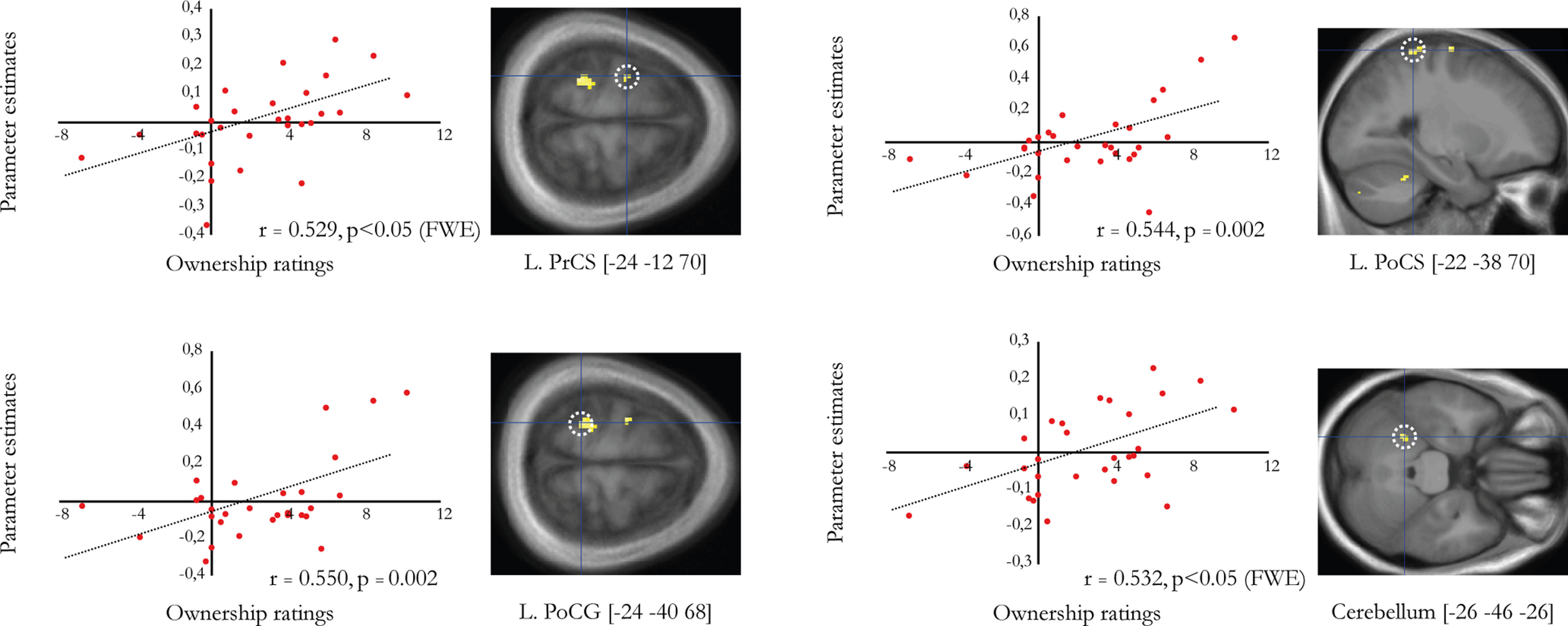
Correlation between behavioral ownership ratings (*x*-axis) and parameter estimates from the ownership contrast (*y*-axis; in a.u.) in the left precentral sulcus (PrCS; −24, −12, 70), left postcentral gyrus (PoCG; −24, −40, 68), left postcentral sulcus (PoCS; −22, −38, 70), and left cerebellum (−26, −46, −26). Pearson's *r* and *p* values are given in each respective correlation plot. The peaks are displayed as activation maps (*p* < 0.005, uncorrected) on representative sections of an average anatomic section and are indicated with a dotted white line.

In a complementary descriptive approach, we followed up on the above ownership interaction contrast by examining whether those BOLD effects also correlated with the subjective ratings in the ownership statements. To this end, we performed a multiple regression analysis using the ownership ratings from each participant to search for voxels whose parameter estimates could be predicted from the behavioral contrast (see Materials and Methods). We identified four such regions whose parameter estimates were significantly correlated with the behavioral contrast (Fig. 6). The activity in the left PMd (−24, −12, 70; *p* < 0.05, Fig. 6) and cerebellum was significant after FWE correction (cerebellum; −26, −46, −26; *p* < 0.05, Fig. 6), whereas the activity in the postcentral gyrus and postcentral sulcus was not (*p* < 0.001, uncorrected; Fig. 6).

### The sense of agency is associated with activity in the left precentral and postcentral gyri as well as right superior temporal gyrus

**Figure 7.**
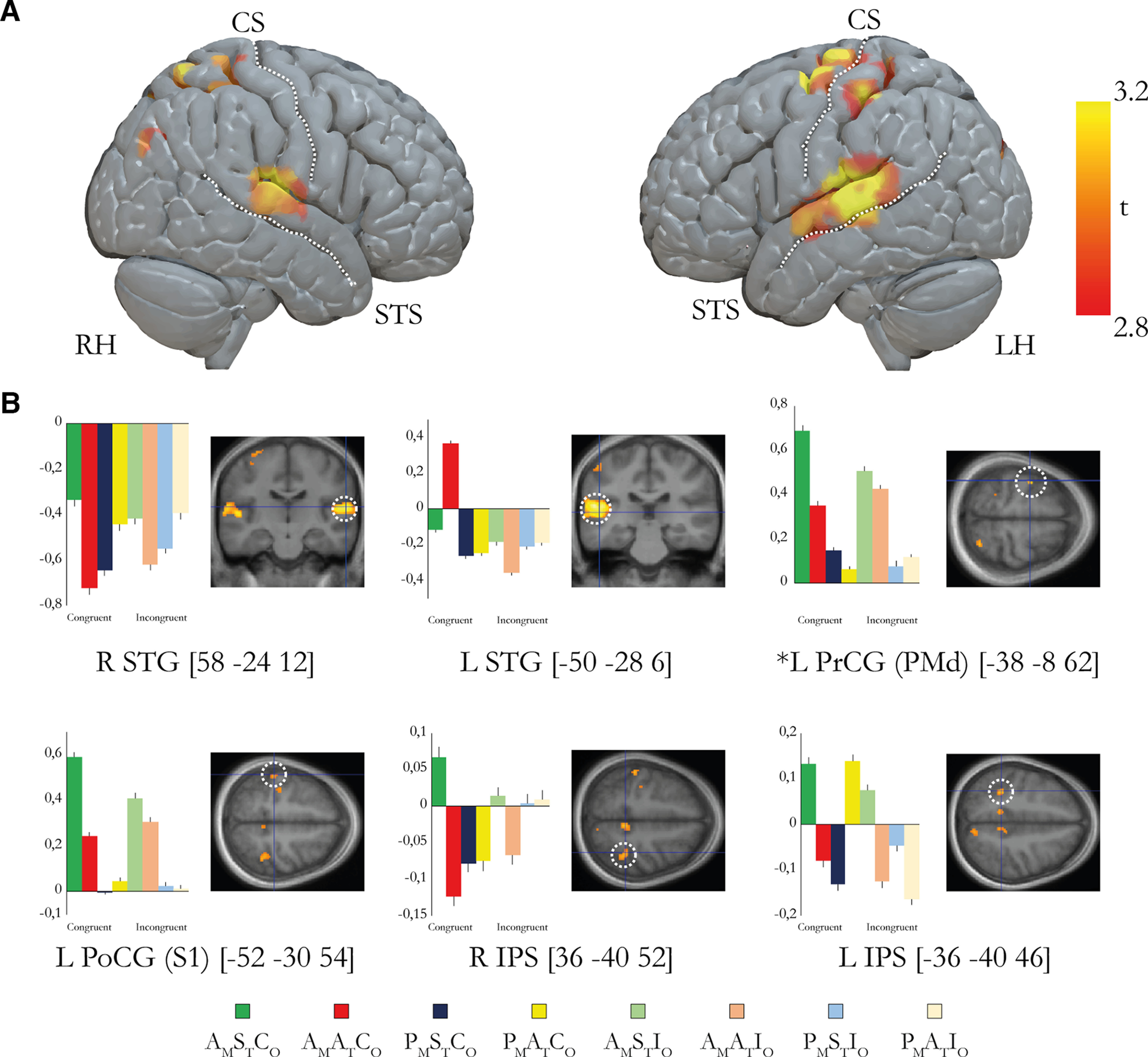
***A***, Overview of the brain regions that display activation reflecting the sense of agency defined by the contrast [(A_M_S_T_C_O_ – P_M_S_T_C_O_) – (A_M_A_T_C_O_ – P_M_A_T_C_O_)] + [(A_M_S_T_I_O_ – P_M_S_T_I_O_) – (A_M_A_T_I_O_ – P_M_A_T_I_O_)]. For display purposes only, the activations are projected onto a three-dimensional render of a standard brain with a threshold of *p* < 0.005 (uncorrected for multiple comparisons, *k* ≥ 5). RH, Right hemisphere; LH, left hemisphere; STS, superior temporal sulcus; CS, central sulcus. ***B***, Bar charts displaying the parameter estimates (in a.u.) and SEs for the major peaks of activation. The coordinates are given in MNI space. The peaks are displayed in representative sections indicated by a dotted white circle (*p* < 0.005, uncorrected for display purposes). L, Left; R, right; PrCG, precentral gyrus; PoCG, postcentral gyrus; IPS, intraparietal sulcus; STG, superior temporal gyrus. *Activation peaks that survive small-volume correction (*p* < 0.05, corrected); the peaks without an asterisk did not survive correction and are reported in Table 2 with their uncorrected *p* value. All peaks from the contrast are reported in Extended Data Table 7-1.

We then examined activations that reflect the sense of agency, that is, increases in activity dependent on actively generated movements as well as synchronous sensory feedback from the moving finger regardless of whether the hand was experienced as part of one's body or not. To this end, we used the contrast [(A_M_S_T_C_O_ – P_M_S_T_C_O_) – (A_M_A_T_C_O_ – P_M_A_T_C_O_)] + [(A_M_S_T_I_O_ – P_M_S_T_I_O_) – (A_M_A_T_I_O_ – P_M_A_T_I_O_)], which represents agency across the congruent and incongruent conditions. In line with our hypotheses, we observed a significant activation peak in the left premotor cortex (−38, −8, 62; *p* < 0.05, FWE corrected; Fig. 7, Table 2) and an activation in the right superior temporal gyrus that almost reached significance (58, −24, 12; *p* = 0.051, FWE corrected; Fig. 7, Table 2). This cluster is the second largest (*k* = 347) in this contrast (the largest one being the left superior temporal gyrus), and its location is very close to the peak from the localizer experiment around which the small volume correction was made, which is why we chose to report it despite the *p* value of 0.051. We also observed increases in activity in the intraparietal cortex bilaterally as well as the left superior temporal gyrus and left postcentral gyrus (*p* < 0.001, uncorrected), but these activations did not survive correction for multiple comparisons and are thus only mentioned for descriptive purposes.

10.1523/JNEUROSCI.1492-22.2023.tab7-1 Table 7-1 All peaks from the ownership contrast *[(A_M_S_T_C_O_-P_M_S_T_C_O_)-(A_M_A_T_C_O_-P_M_A_T_C_O_)] + [(A_M_S_T_I_O_-P_M_S_T_I_O_)-(A_M_A_T_I_O_-P_M_A_T_I_O_)]*. All peaks that survived the threshold p<0.005 (uncorrected, k≥10) are reported. Download Table 7-1, DOCX file.

### Agency and ownership overlap in the precentral gyrus

**Figure 8.**
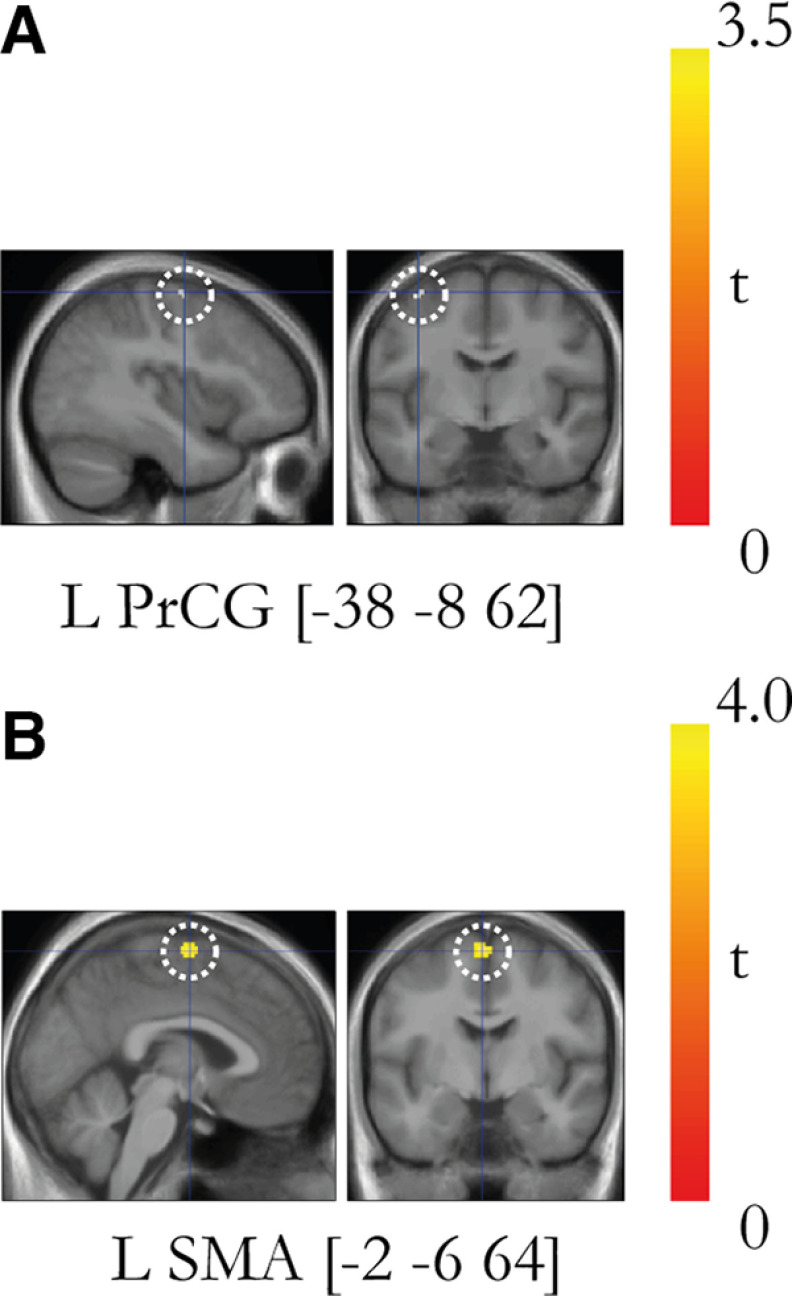
***A***, Conjunction analysis between the agency contrast and ownership contrast revealed overlapping activation in the left PMd. The significant activation peak (*p* < 0.05, corrected) is displayed on a representative section (*p* < 0.005, uncorrected) and is indicated with a dotted white line. ***B***, PPI analysis of regions displaying increased connectivity with the seed region in the left postcentral gyrus (−38, −28, 52). The left SMA displays a task-specific increase in connectivity with the left postcentral gyrus (SMA; *t* = 3.56; *p* = 0.001, uncorrected). The peak is displayed as part of an activation map (*p* < 0.005, uncorrected) and is indicated with a dotted white line. The activation maps are presented on representative sagittal and coronal sections of a mean anatomic MRI image made up of all participants' structural brain scans.

To test for areas that showed increases in activity reflecting both ownership and agency, we used a conjunction analysis with the two two-way interaction contrasts described above for ownership and agency (Friston et al., 1999; Fig. 8*A*). The analysis revealed a significant activation peak in the precentral gyrus (PMd, −38, −8 62; *p* < 0.05, FWE corrected; Fig. 8*A*).

### Interaction between ownership and agency revealed activation in the somatosensory cortex

**Figure 9.**
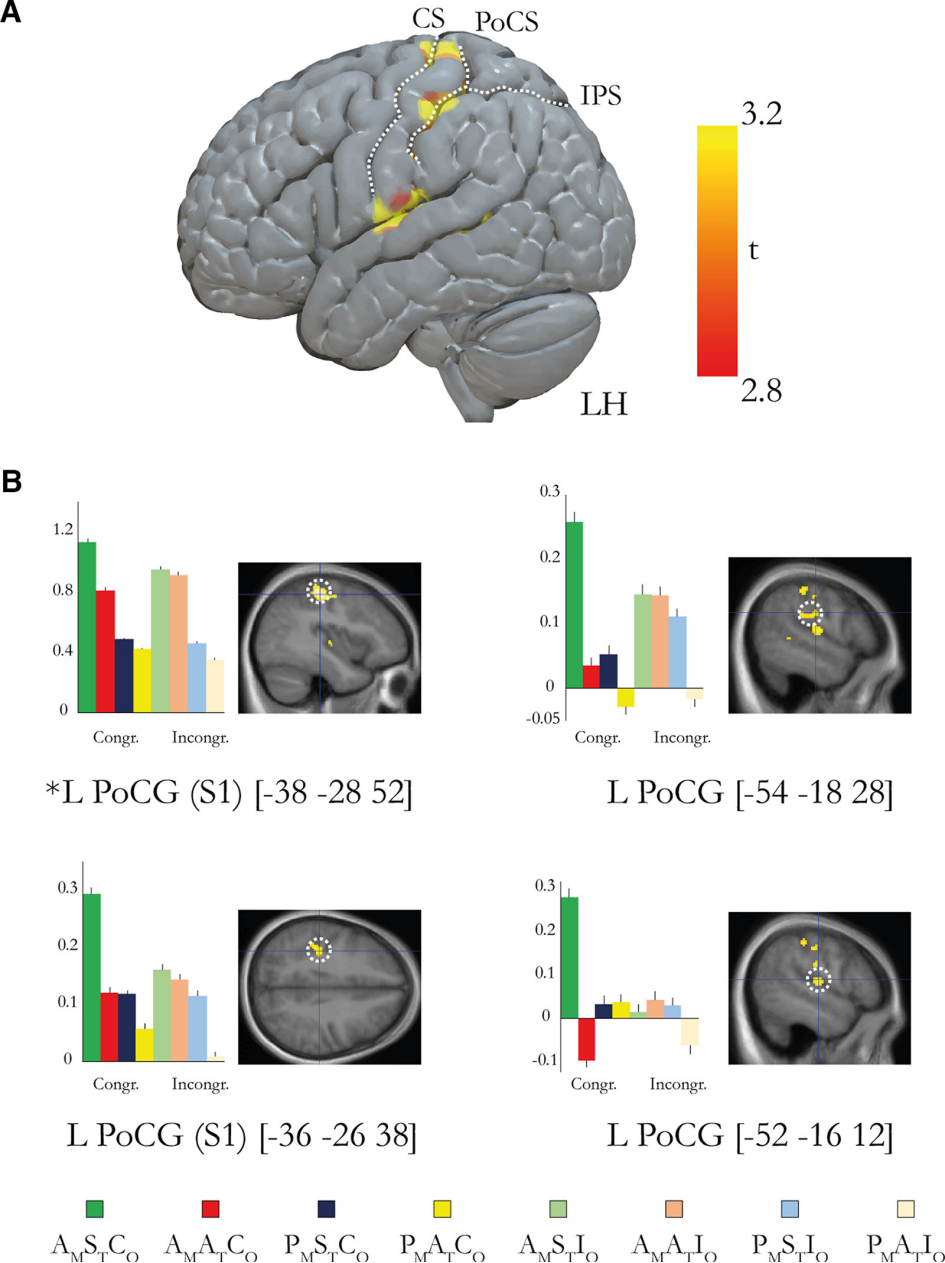
***A***, Overview of the brain regions that display activation reflecting the unique combination of agency and body ownership as defined by the contrast [(A_M_S_T_C_O_ – P_M_S_T_C_O_) – (A_M_A_T_C_O_ – P_M_A_T_C_O_)] – [(A_M_S_T_I_O_ – P_M_S_T_I_O_) – (A_M_A_T_I_O_ – P_M_A_T_I_O_)]. For display purposes only, the activations are projected onto a three-dimensional rendering of a standard brain with a threshold of *p* < 0.005 (uncorrected for multiple comparisons, *k* ≥ 5). RH, Right hemisphere; LH, left hemisphere; IPS, intraparietal sulcus; PoCS, postcentral sulcus; CS, central sulcus. ***B***, Bar charts displaying the parameter estimates (in a.u.) and SEs for the major peaks of activation. The coordinates are given in MNI space. The peaks are displayed in representative sections indicated by a dotted white circle on an activation map (*p* < 0.005, uncorrected for display purposes). L, left; R, right; PoCG, postcentral gyrus. *Activation peaks that survive small-volume correction (*p* < 0.05 corrected); the peaks without an asterisk did not survive small-volume correction and are reported in Table 2 with their uncorrected *p* value. All peaks from the contrast are reported in Extended Data Table 9-1. Condition key: first letter A or P (active or passive) with subscript M (movement); second letter S or A (synchronous or asynchronous) with subscript T (timing); third letter C or I (congruent or incongruent) with subscript O (orientation).

To test for interaction between ownership and agency, we used the contrast [(A_M_S_T_C_O_ – P_M_S_T_C_O_) – (A_M_A_T_C_O_ – P_M_A_T_C_O_)] – [(A_M_S_T_I_O_ – P_M_S_T_I_O_) – (A_M_A_T_I_O_ – P_M_A_T_I_O_)]. This corresponds to the three-way interaction among movement type (active/passive), timing (synchronous/asynchronous), and rubber hand orientation (congruent/incongruent), and thus reveals neural responses unique to the combination of ownership and agency in the moving rubber hand illusion condition (A_M_S_T_C_O_). The results show significant activation in the left primary sensorimotor cortex with a significant peak of activation located in the postcentral gyrus at the level of the hand representations (−38, −28, 52; *p* < 0.05, FWE corrected; Fig. 9) and three further peaks in the postcentral gyrus that did not survive corrections for multiple comparisons (*p* < 0.005; Fig. 9, Table 2).

10.1523/JNEUROSCI.1492-22.2023.tab9-1 Table 9-1 All peaks from the ownership x agency interaction contrast *[(A_M_S_T_C_O_-P_M_S_T_C_O_)-(A_M_A_T_C_O_-P_M_A_T_C_O_)]-[(A_M_S_T_I_O_-P_M_S_T_I_O_)-(A_M_A_T_I_O_-P_M_A_T_I_O_)]*. All peaks that survived the threshold p<0.005 (uncorrected, k≥10) are reported. Download Table 9-1, DOCX file.

10.1523/JNEUROSCI.1492-22.2023.tab10-1 Table 10-1 All peaks from the inverse of the ownership x agency interaction contrast *[(A_M_S_T_C_O_-P_M_S_T_C_O_)-(A_M_A_T_C_O_-P_M_A_T_C_O_)]-[(A_M_S_T_I_O_-P_M_S_T_I_O_)-(A_M_A_T_I_O_-P_M_A_T_I_O_)]*. All peaks that survived the threshold p<0.005 (uncorrected, k≥10) are reported. Download Table 10-1, DOCX file.

We should clarify here that the somatosensory activation under discussion can probably not be explained by somatosensory attenuation (Zeller et al., 2015; Kilteni and Ehrsson, 2017, 2020) or gating (Angel and Malenka, 1982; Post et al., 1994; Voudouris et al., 2019; Kilteni and Ehrsson, 2022) because we observed an increase in activity, not a reduction. Moreover, we controlled the amplitude of the movements, and there were no significant differences in movement frequency between conditions (see below; Fig. 13). Therefore, it is unlikely that low-level differences in motor output or somatosensory feedback confounded our S1 findings. We also think it is implausible that differences in tap force between the active and passive movements could explain our results because participants were trained to apply gentle taps and the experimenter reproduced such gentle taps in the passive condition; furthermore, the effect of active versus passive movements are matched in the three-way interaction contrast (as well as in the agency and ownership interaction contrasts).

**Figure 10.**
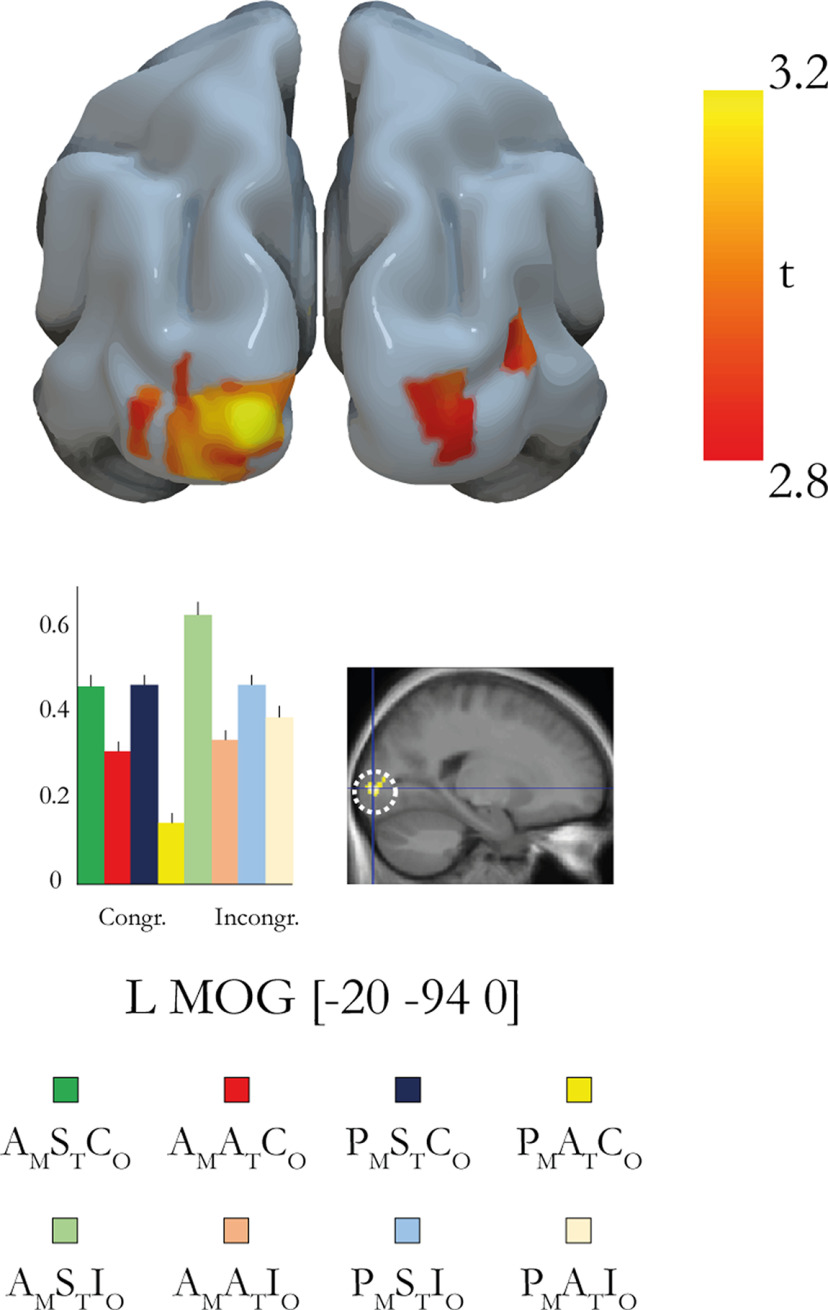
To investigate which brain regions are associated with the sense of agency of external objects as opposed to bodily objects, we defined a contrast that was the inverse of the three-way interaction [(A_M_S_T_C_O_ – P_M_S_T_C_O_) – (A_M_A_T_C_O_ – P_M_A_T_C_O_)] – [(A_M_S_T_I_O_ – P_M_S_T_I_O_) – (A_M_A_T_I_O_ – P_M_A_T_I_O_)]. The results show activation in the left middle occipital gyrus (*p* < 0.001, uncorrected; did not survive correction for multiple comparisons) and right middle occipital gyrus (*p* = 0.002, uncorrected). The coordinates are given in MNI space. L, left; R, right; MOG, middle occipital gyrus. The peak is displayed in a representative section and indicated by a dotted white circle on an activation map (*p* < 0.005, uncorrected for display purposes; *k* ≥ 5). The bar chart represents the parameter estimates (in a.u.) for the peak.

Next, we examined the opposite direction of the three-way interaction contrast of movement type, synchrony, and orientation [(A_M_S_T_C_O_ – P_M_S_T_C_O_) – (A_M_A_T_C_O_ – P_M_A_T_C_O_)] – [(A_M_S_T_I_O_ – P_M_S_T_I_O_) – (A_M_A_T_I_O_ – P_M_A_T_I_O_)]. This contrast revealed only one activation in the left middle occipital gyrus and one smaller activation in the right middle occipital gyrus (Fig. 10, Table 2), but neither of these activations survived correction for multiple comparisons.

### Psychophysiological interaction analysis of functional connectivity

Our results reported above revealed activation in the postcentral gyrus (S1) associated with the combined experience of illusory ownership and agency (three-way interaction). This made us curious if there could be changes in functional connectivity between S1 and other brain areas that could help us understand this finding further. Thus, in a *post hoc* exploratory PPI analysis of the functional connectivity in the three-way interaction of the factors timing, movement type, and orientation, we investigated the task-specific connectivity changes between the section of the postcentral gyrus under discussion (−38, –28, 52) and the rest of the brain. We found that the sense of ownership in the presence of a sense of agency increased the functional coupling between the left primary sensory cortex and the ipsilateral SMA (−2, −6, 64; *t* = 3.56; *p* = 0.001, uncorrected; Fig. 8*B*). In the rest of the brain, no active clusters were observed apart from one in cerebellum (R VIIb; 28, −68, −46; *t* = 3.51; *p* = 0.001, uncorrected).

### Activations in the insular cortex and right temporoparietal cortex reflect visuoproprioceptive synchrony and asynchrony, respectively

**Figure 11.**
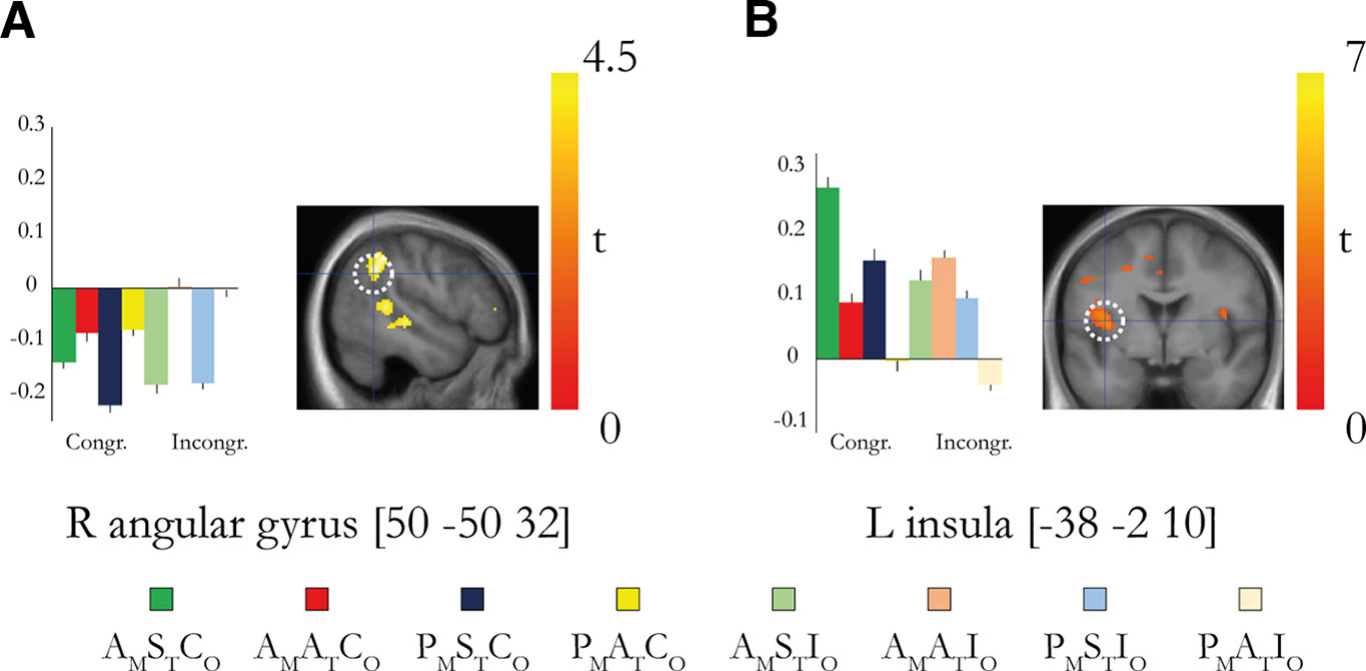
***A***, Activation in the right angular gyrus represented by the main effect of asynchrony: (A_M_A_T_C_O_ + P_M_A_T_C_O_ + A_M_A_T_I_O_ + P_M_A_T_I_O_) – (A_M_S_T_C_O_ + P_M_S_T_C_O_ + P_M_S_T_I_O_ + A_M_S_T_I_O_). ***B***, Activation in the left insular cortex represented by the main effect of synchrony: (A_M_S_T_C_O_ + P_M_S_T_C_O_ + P_M_S_T_I_O_ + A_M_S_T_I_O_) – (A_M_A_T_C_O_ + P_M_A_T_C_O_ + A_M_A_T_I_O_ + P_M_A_T_I_O_). The coordinates are given in MNI space. The peak is displayed in a representative section and is indicated by a dotted white circle on an activation map (*p* < 0.005, uncorrected for display purposes).

In the previous literature, it has been suggested that the right angular gyrus located in the temporoparietal region is involved in the loss of agency when there is a mismatch between the expected sensory consequences of self-generated movement and the sensory feedback (Farrer and Frith, 2002; Farrer et al., 2003; Tsakiris et al., 2010). Furthermore, it has been reported that the insular cortex shows increases in activation when people experience agency (Farrer and Frith, 2002; Farrer et al., 2003). However, in our main planned contrasts reported above, we did not find any changes in activation in these two regions, even at the level of uncorrected *p* values (*p* < 0.005). To examine this apparent inconsistency further, we looked at the main effect of synchrony [(A_M_S_T_C_O_ + A_M_S_T_I_O_ + P_M_S_T_C_O_ + P_M_S_T_I_O_) – (A_M_A_T_C_O_ + A_M_A_T_I_O_ + P_M_A_T_C_O_ + P_M_A_T_I_O_)] and the main effect of asynchrony contrasts [(A_M_A_T_C_O_ + P_M_A_T_C_O_ + A_M_A_T_I_O_ + P_M_A_T_I_O_) – (A_M_S_T_C_O_ + P_M_S_T_C_O_ + P_M_S_T_I_O_ + A_M_S_T_I_O_); i.e., areas that show greater activation when visual feedback and finger movements are synchronous or asynchronous regardless of the senses of ownership or agency (i.e., across active and passive movements and across anatomically congruent or incongruent hand orientations)]. Interestingly, we found a large and significant activation (*t* = 3.66; *p* = 0.022, FWE corrected) located in the right angular gyrus of the TPJ region (50, −50, 32) that reflected the asynchronous relation between movement and visual feedback (main effect of asynchrony; Fig. 11*A*). In contrast, the synchrony of finger movements and visual feedback of the finger movement of the model hand (main effect of synchrony) was associated with significant activation (*t* = 3.71; *p* = 0.020, FWE corrected) of the left insular cortex (−38, −2, 10; Fig. 11*B*). Thus, rather than reflecting the sense of agency or the loss of agency by mismatching sensory feedback, our results suggest that the insular cortex and right temporoparietal cortex are involved in the basic detection of synchronous or asynchronous multimodal stimuli.

### Activation in the supplementary motor cortex reflects the main effect of active versus passive movements

**Figure 12.**
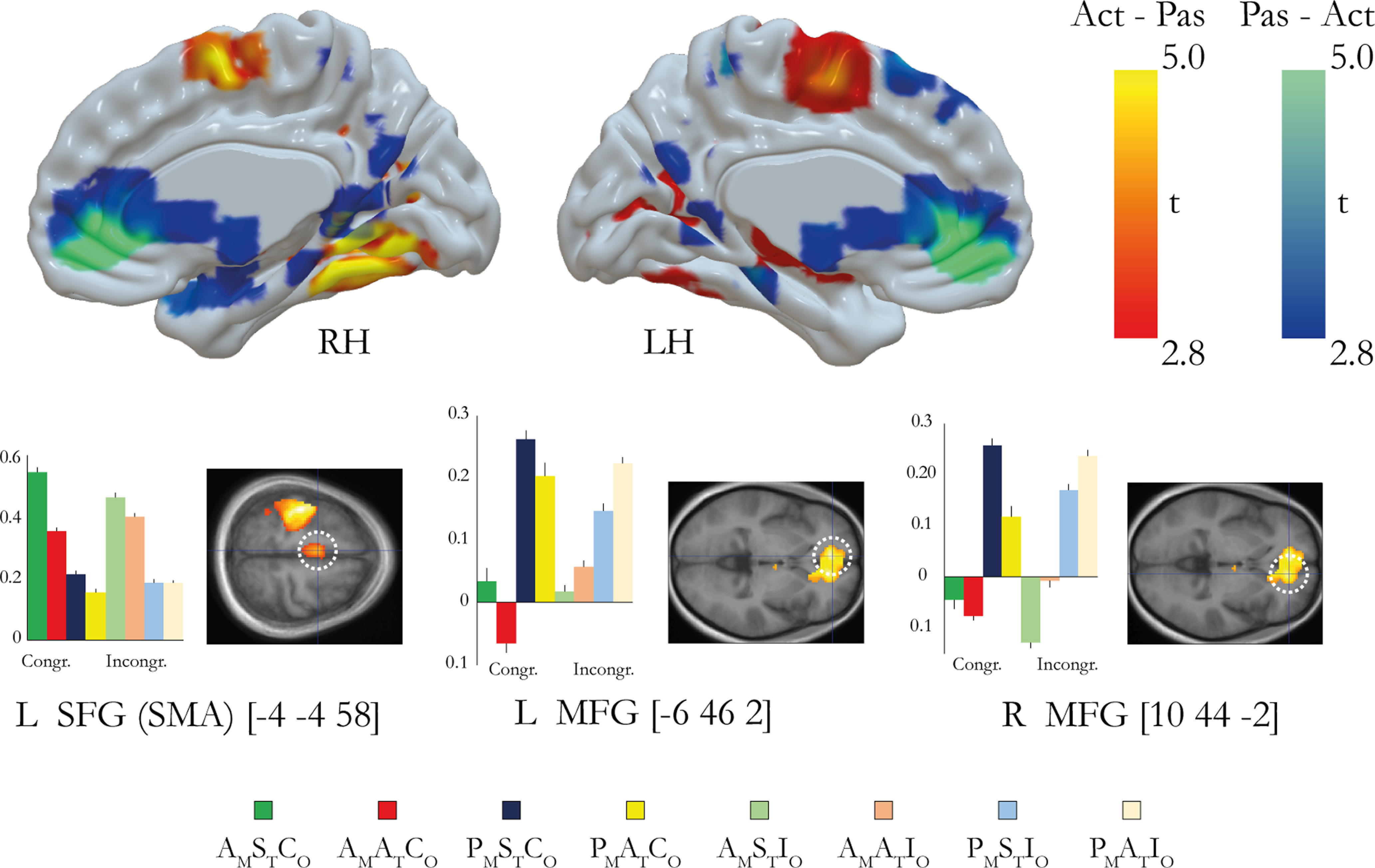
Main effect of movement type (active or passive). Using the contrast (A_M_S_T_C_O_ + A_M_A_T_C_O_ + A_M_S_T_I_O_ + A_M_A_T_I_O_) – (P_M_S_T_C_O_ + P_M_A_T_C_O_ + P_M_S_T_I_O_ + P_M_A_T_I_O_), we compared all active movement conditions to all passive conditions (regardless of ownership or agency; yellow–red color scale for activation; top row). Active movement was associated with significant activations in the left supplementary motor area (−4, −4, 58; *t* = 4.98; *p* < 0.001 uncorrected), left precentral gyrus (PMd; −42, −10, 60; *t* = 7.82; *p* < 0.001, FDR corrected; data not shown), left precentral gyrus (M1; −40, −18, 56; *t* = 9.20; *p* < 0.011, FDR corrected; data not shown), right cerebellum (lobule VI; 20, −50, −24; *t* = 9.23; *p* < 0.001, FDR corrected; data not shown), left thalamus (−14, −22, 4; *t* = 5.90; *p* = 0.026, FDR corrected; data not shown), and right angular gyrus (34, −50, 24; *t* = 5.79; *p* = 0.033, FDR corrected; data not shown). We also compared all passive movement conditions to all active movement conditions, (P_M_S_T_C_O_ + P_M_A_T_C_O_ + P_M_S_T_I_O_ + P_M_A_T_I_O_) – (A_M_S_T_C_O_ + A_M_A_T_C_O_ + A_M_S_T_I_O_ + A_M_A_T_I_O_). Passive movements were associated with a relative increase in neural activity compared with active movements in the bilateral medial frontal cortex (only right shown in section: 10, 44, −2; *t* = 5.8; *p* < 0.001, uncorrected; left medial frontal cortex: −6, 46, −2; *t* = 5.18; *p* < 0.001; blue–green color scale for activation). The peaks are displayed in a representative section and are indicated by a dotted white circle on an activation map (*p* < 0.005, uncorrected for display purposes). All peaks from the contrast are reported in Extended Data Table 12-1. RH, Right hemisphere; LH, left hemisphere; SFG, superior frontal gyrus; MFG, medial frontal gyrus.

Another area suggested to be involved in agency in previous fMRI studies, including agency in the moving RHI (Tsakiris et al., 2010), is the SMA. However, this area did not show any agency-related activity in our agency contrast described above, not even at *p* < 0.005 uncorrected. However, when we examined the main effect of movement type, contrasting all active movement versus all passive movement conditions in the current design, we observed significant activation of the SMA (A_M_S_T_C_O_ + A_M_A_T_C_O_ + A_M_S_T_I_O_ + A_M_A_T_I_O_) – (P_M_S_T_C_O_ + P_M_A_T_C_O_ + P_M_S_T_I_O_ + P_M_A_T_I_O_; Fig. 12). This region seems to be important for generating movements voluntarily, thereby indicating its role movement planning, programming, and volition more generally (Roland et al., 1980; Fried et al., 1991; Makoshi et al., 2011). However, we found no evidence for specific involvement in the sense of agency of the moving rubber hand.

10.1523/JNEUROSCI.1492-22.2023.tab12-1 Table 12-1 The peaks from the localizer experiment used to define the ROIs in the current study. Download Table 12-1, DOCX file.

When we looked for areas showing greater activity in the passive movement conditions than in the active ones, we found a large activation in the medial prefrontal cortex in a region associated with default mode activity (Raichle et al., 2001; Buckner et al., 2008; Tacikowski et al., 2017), autobiographical episodic memory (Maguire, 2001; Svoboda et al., 2006; Bergouignan et al., 2014), and self-related information processing (Qin and Northoff, 2011; Tacikowski et al., 2017). The most straightforward interpretation is that since participants did not have an active task in this condition (they just relaxed their hand, and the experimenter generated the finger movements), the activity was higher in the default mode, thus explaining the relatively higher activity in this medial prefrontal region compared with the active movement conditions when the participant had a task to move their finger repeatedly. This activation also corresponds well to similar activity observed in the passive finger movement condition in the study of Tsakiris et al. (2010), which these authors attributed to ownership (Fig. 12).

### Controlling for the number and frequency of taps in the different conditions

**Figure 13.**
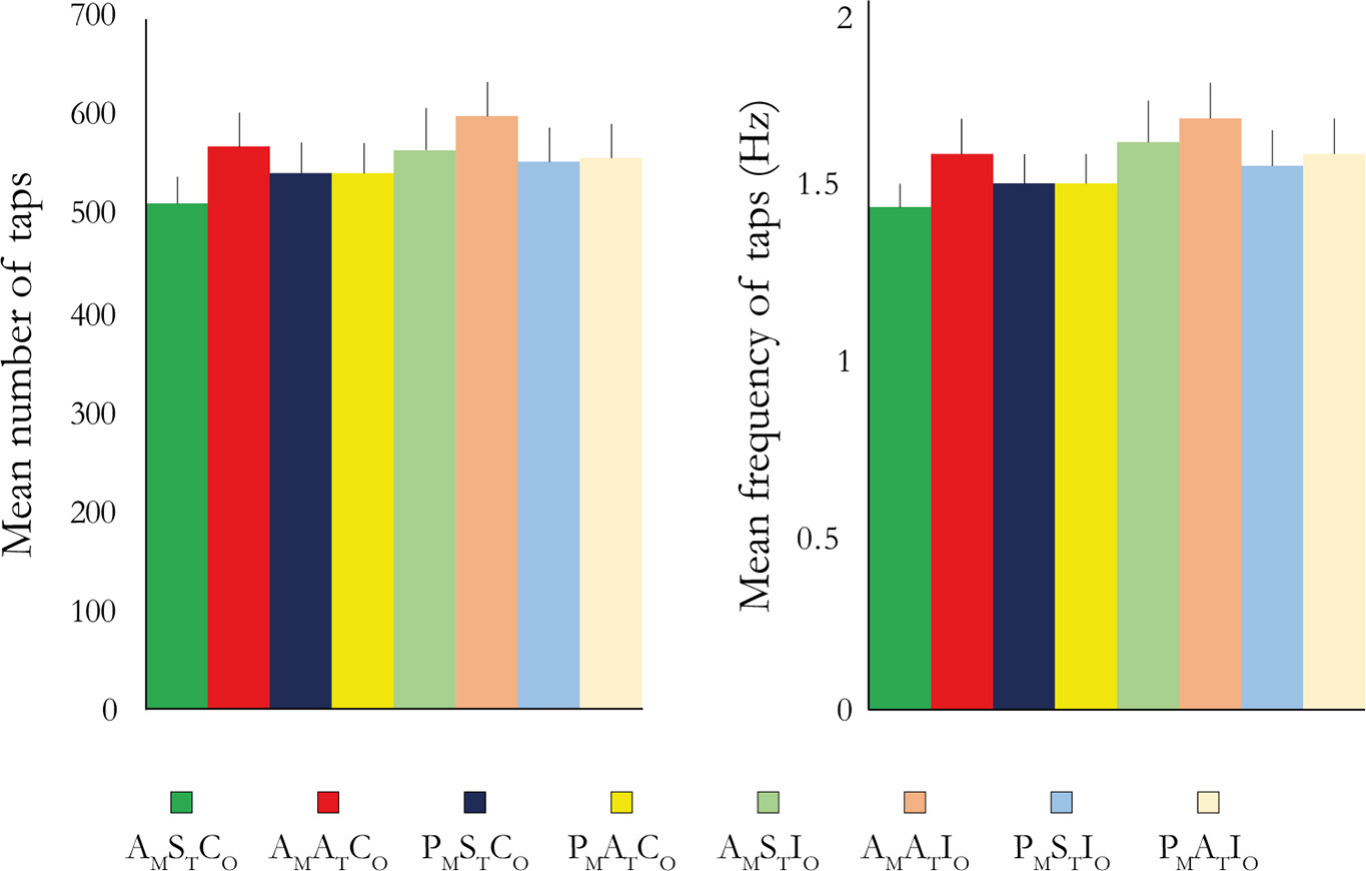
The number and frequency of taps across conditions. The bars represent the mean number and frequency of taps for all conditions for the period excluding the illusion onset times (see Materials and Methods). Error bars indicate the SEMs. The analysis of the frequencies of taps revealed no significant main effects and no significant interactions, and there were no differences in frequencies across conditions. The exact values for each condition are given in Extended Data Table 13-1.

Using the optical sensor placed under the index finger of the participants, the number of taps as well as the frequency of taps for each condition could be analyzed. The analysis was performed on the time periods included in the fMRI analysis (i.e., excluding the time before illusion onset and the corresponding time periods for conditions without illusion). A one-way ANOVA revealed no significant differences across conditions for the frequency of taps (mean, 1.53 Hz; *F* = 0.636; df = 7; *p* = 0.725; Fig. 13). Moreover, when the frequencies of taps were analyzed using the same 2 × 2 × 2 design as the fMRI experiment, we found no significant main effect of movement type (*F* = 2.519; df = 19, 1; *p* = 0.129; η^2^ = 0.014), no significant main effect of timing (*F* = 2.353; df = 19, 1; *p* = 0.142; η^2^ = 0.007), no significant main effect of orientation (*F* = 2.390; df = 19, 1; *p* = 0.139; η^2^ = 0.041), and no significant interactions (movement type × timing: *F* = 0.928; df = 19,1; *p* = 0.348, η^2^ = 0.008; movement type × orientation: *F* = 0.152; df = 19,1; *p* = 0.701; η^2^<0.001; orientation × timing: *F* = 2.215; df = 19,1; *p* = 0.152; η^2^ = 0.006; movement type × timing × orientation: *F* = 0.430; df = 19,1; *p* = 0.520; η^2^ = 0.003).

## Discussion

This study has three main novel findings. First, the neural substrates of ownership and agency were largely distinct, with body ownership associated with increases in activity in the premotor cortex, posterior parietal, and cerebellar regions, and the sense of agency related to increased activity in the superior temporal cortex and dorsal premotor cortex. Second, one active section of the dorsal premotor cortex was associated with both agency and body ownership, indicating a cortical site where ownership and agency information may be combined. Third, there was an interaction between body ownership and agency in the somatosensory cortex so that its activity was higher when participants experienced both sensations. This was accompanied by higher ownership ratings, suggesting an agency-induced ownership enhancement of somatosensory cortical activity specific for voluntary movement. Collectively, these findings extend our knowledge of the neural basis of body ownership and agency, and reveal their functional interaction and the relative neuroanatomical overlap and segregation during simple movement, which advances our understanding of how bodily self-consciousness is implemented in the human brain.

### The sense of body ownership during movement: integration of spatiotemporally congruent visuoproprioceptive signals in premotor-parietal-cerebellar regions

The present study extends the previous neuroimaging literature on the neural basis of body ownership (Ehrsson et al., 2004; Petkova et al., 2011; Brozzoli et al., 2012; Gentile et al., 2013; Guterstam et al., 2013, 2019; Limanowski and Blankenburg, 2016; Preston and Ehrsson, 2016; Chancel et al., 2022b) into such experience arising from the sensory feedback of movement. The sense of ownership of the moving rubber hand was associated with significant activations in the left premotor cortex (precentral gyrus), posterior parietal cortex (left supramarginal gyrus), and right lateral cerebellum. These activations probably reflect the integration of spatially and temporally congruent visual information from the moving rubber hand and kinesthetic–proprioceptive information from the hidden real hand because the neural response was specifically related to the conditions when the rubber hand was placed in an anatomically congruent condition and the seen and felt movements were synchronous (i.e., when the visual and kinesthetic–proprioceptive information obeyed the temporal and spatial rules of body ownership; Ehrsson, 2012; Kalckert and Ehrsson, 2012; Blanke et al., 2015; Samad et al., 2015; Chancel et al., 2022a), controlling for agency effects and effects related to active versus passive movement.

The difference between visuokinesthetic integration, which was studied herein, and visuotactile integration, which was investigated in previous RHI studies, can probably explain the differences in precise localization of the activation peaks in the premotor cortex compared with a previous study (Ehrsson et al., 2004). Although activations have been seen in both ventral and dorsal aspects of the premotor cortex in previous RHI studies (Gentile et al., 2013; Guterstam et al., 2019), the most consistent activations tend to have been located in the ventral premotor cortex (Ehrsson et al., 2004; Gentile et al., 2013; Guterstam et al., 2013, 2019; Limanowski and Blankenburg, 2016; Grivaz et al., 2017). The dorsal premotor cortex is active during passive hand and arm movements (Zhavoronkova et al., 2017), finger tapping (Ullén et al., 2003; Bengtsson et al., 2009), and illusory hand and arm movements triggered by muscle tendon vibration (Naito et al., 1999, 2005), which is consistent with a role in multisensory representation of the upper limb in space. The current activation in the SMG (*p* < 0.05, corrected) is consistent with the findings of earlier body ownership illusion studies based on visuotactile stimulation (Gentile et al., 2013; Petkova et al., 2011), and the current intraparietal cortex activation is located in a section of this sulcus associated with multisensory integration in perihand space (Lloyd et al., 2003; Makin et al., 2007; Brozzoli et al., 2011) and illusory hand ownership (Chancel et al., 2022b). We also observed activity in the ipsilateral lateral cerebellum that is in line with previous fMRI studies on various versions of the rubber hand illusion based on visuotactile stimulation (Ehrsson et al., 2004, 2005; Guterstam et al., 2013) and limb movement illusions (Ehrsson et al., 2005; Hagura et al., 2009). Importantly, the current findings extend the previous literature on body ownership and body representation by demonstrating a role for these premotor-parietal-cerebellar regions in the sense of limb ownership during movement.

### The sense of agency in one's own bodily movement: premotor and superior temporal cortex

We could isolate activity in the dorsal premotor cortex and superior temporal cortex reflecting agency over limb movement while controlling for unspecific effects related to multisensory synchrony–asynchrony detection, active versus passive movement, and body ownership. The dorsal premotor area has been reported in previous studies on the sense of agency over sensory events caused by voluntary movement (David et al., 2008; Yomogida et al., 2010; Nahab et al., 2011; Sperduti et al., 2011; Haggard, 2017), so our finding extends this to agency over perceived own bodily movement. The dorsal premotor cortex is anatomically connected to and receives input from the dorsolateral prefrontal cortex regarding intentions and the initiation of voluntary action in the context of an overall action plan (Passingham, 1993; Koechlin et al., 2003; Abe and Hanakawa, 2009; Yamagata et al., 2012) and receives multisensory input from the posterior parietal cortex regarding one's own body as well as external sensory events; the dorsal premotor area can also influence movement execution in M1 and receive feedback from this area through direct corticocortical connections (Porter and Lemon, 1995; Dum et al., 2002). The dorsal premotor cortex is thus in an excellent position, anatomically and physiologically, to play a central role in the sense of agency by integrating and comparing signals related to voluntary motor commands and sensory feedback, consistent with our findings.

Interestingly, the section of the dorsal premotor cortex associated with agency also showed body ownership-related activity, as revealed in our conjunction analysis. This finding suggests that the neural bases of body ownership and agency are not completely distinct (Tsakiris et al., 2010), and that least one cortical area is involved in both processes. Different neuronal populations within the dorsal premotor cortex could implement the formation of a coherent multisensory representation of the hand in space (ownership), the generation of voluntary motor commands, and the matching of the outcomes of those commands with the sensory feedback and predictions (agency) or the same neuronal population within this area may implement both of these mechanisms (which could be tested in future studies with BOLD adaptation or multivoxel pattern analysis). Our findings suggest a more intimate relationship of the representations of body ownership and agency in the premotor cortex than commonly assumed and indicate that more attention should be devoted to this region in future studies on the neural mechanisms of agency of bodily action.

Previous neuroimaging studies have suggested that the superior temporal cortex plays a role in the sense of agency, but they reported that activation in the superior temporal gyrus reflected the loss of agency when controlling a virtual limb (Nahab et al., 2011; Uhlmann et al., 2020). However, these studies did not control for multisensory synchrony–asynchrony, the visual appearance (and identity) of the hand, or body ownership. In contrast, we found a relative activity increase that reflected gaining agency of the moving rubber hand, although all experimental conditions were deactivated compared with the resting baseline. The current activation peak is located more ventral and anterior to the deactivations in previous studies (Nahab et al., 2011; Uhlmann et al., 2020), making direct comparisons difficult. Although the precise functional role of the superior temporal cortex in agency is unclear, this region has been associated with action observation (Kilintari et al., 2014), visual processing of biological motion (Saygin, 2007), and perception of causality between sensory events (Blakemore et al., 2001), which collectively point toward a function of supporting the (visual) perception of causality relationships between the seen finger movement and the executed finger action, which presumably is an important component of the agency experience.

### Interaction of body ownership and agency in the somatosensory cortex

Our analysis revealed somatosensory activity that was uniquely related to the situation when both ownership and agency were experienced over the moving rubber hand (interaction between ownership and agency). In principle, this activity could reflect a change in body ownership caused by agency or a change in agency caused by ownership. We think the former is more likely because the behavioral data showed a significant corresponding interaction effect in the questionnaire hand ownership ratings but not in the agency ratings. Thus, the somatosensory activity may be related to a change in the somatic feeling of the rubber hand illusion when this illusion is produced by visuomotor–kinesthetic correlations during active movements as opposed to visuokinesthetic correlations during passive movements. Motor commands and efferent signals can influence limb movement sensations (Gandevia et al., 2006; Walsh et al., 2010), and thus, we theorize that information related to the active motor command signals made the ownership experience more vivid by boosting kinesthetic sensations from the finger movements of the rubber hand. Such motor command signals could originate from premotor areas and influence the somatosensory cortex via corticocortical connections, which is supported by the finding of increased functional connectivity between the SMA and S1 in the active synchronous congruent condition when both ownership and agency were experienced (Fig. 8). Alternatively, agency might influence the multisensory integration process that determines body ownership by facilitating combination over segregation by influencing the prior probability of a common cause (Samad et al., 2015; Chancel et al., 2022a), although it remains unclear how this would lead to enhanced S1 activation rather than increased premotor or posterior parietal activity. The somatosensory activity might also reflect a special component of agency over one's bodily movements—“bodily agency”—perhaps reflecting differences between own movement-related somatosensory predictions and predictions about external (e.g., visual) events that are indirectly caused by voluntary action (Frith et al., 2000). According to this view, somatosensory activity would reflect somatosensory predictions during bodily agency, whereas visual cortical activity would reflect visual predictions associated with “external agency” over the nonowned (rotated) rubber hand (Fig. 10). Regardless of the underlying mechanism and conceptualization, our finding links somatosensory activity to the combination of ownership and agency during voluntary limb movement.
