# Supplementary material for: Neural Substrates of Body Ownership and Agency during Voluntary Movement
Source: J Neurosci. 2023 Mar 29;43(13):2362–80. doi: 10.1523/JNEUROSCI.1492-22.2023 (PMC10072298; doi:10.1523/JNEUROSCI.1492-22.2023)
Supplement: Table 3-1 — Illusion onset time measurements for each participant in both the AMSTCO and PMSTCO conditions. Download Table 3-1, DOCX file. [file ns-JN-RM-1492-22-s01.docx]

Table 3-1. Illusion onset time measurements for each participant in both the A_M_S_T_C_O_ and P_M_S_T_C_O_ conditions.

| **Time to illusion onset (s)** | | | |
| --- | --- | --- | --- |
| Participant no | Active/Synchronous/Congruent | Passive/Synchronous/Congruent | Mean |
| 1 | 0 | 0 | 0 |
| 2 | 5.6 | 11.9 | 8.75 |
| 3 | 21.11 | 27.42 | 24.265 |
| 4 | 11.16 | 9.28 | 10.22 |
| 5 | 19.96 | 29.95 | 24.955 |
| 6 | 15.93 | 29.09 | 22.51 |
| 7 | 5.43 | 8.8 | 7.115 |
| 8 | 4.2 | 2.66 | 3.43 |
| 9 | 3.9 | 5.6 | 4.75 |
| 10 | 7.6 | 8.3 | 7.95 |
| 11 | 23.6 | 13.1 | 18.35 |
| 12 | 13 | 12.3 | 12.65 |
| 13 | 0 | 0 | 0 |
| 14 | 22.4 | 7.4 | 14.9 |
| 15 | 30 | 30 | 30 |
| 16 | 7.8 | 20.8 | 14.3 |
| 17 | 15 | 11.1 | 13.05 |
| 18 | 18.7 | 8.1 | 13.4 |
| 19 | 5.2 | 6 | 5.6 |
| 20 | 0 | 0 | 0 |
| 21 | 7.2 | 13.9 | 10.55 |
| 22 | 7.5 | 10.4 | 8.95 |
| 23 | 22.3 | 13.5 | 17.9 |
| 24 | 14.7 | 10.2 | 12.45 |
| 25 | 22.6 | 30.2 | 26.4 |
| 26 | 10.6 | 12.5 | 11.55 |
| 27 | 5 | 7.4 | 6.2 |
| 28 | 7.58 | 9.5 | 8.54 |
| 29 | 5.6 | 6.4 | 6 |

Table 4-1. Mean ratings and standard deviation of each statement from the behavioral experiment.

| **Statement** | **Mean per condition** | | | | | | | | **Standard deviation per condition** | | | | | | | |
| --- | --- | --- | --- | --- | --- | --- | --- | --- | --- | --- | --- | --- | --- | --- | --- | --- |
|  | ASC | AAC | PSC | PAC | ASI | AAI | PSI | PAI | ASC | AAC | PSC | PAC | ASI | AAI | PSI | PAI |
| I felt as if I was looking at my own hand | 1.44 | -0.93 | 0.74 | -0.70 | -1.74 | -2.00 | -1.26 | -2.00 | 1.85 | 2.17 | 1.76 | 2.26 | 1.79 | 1.52 | 2.04 | 1.55 |
| I felt as if the rubber hand was part of my body | 1.15 | -0.89 | 0.52 | -0.33 | -1.96 | -1.96 | -1.56 | -2.07 | 1.88 | 2.29 | 1.89 | 2.19 | 1.48 | 1.33 | 1.53 | 1.38 |
| It seemed as if I were sensing the movement of  my finger in the location where the rubber finger moved | 1.37 | -0.63 | 1.15 | -0.78 | -1.19 | -1.41 | -0.37 | -1.59 | 1.70 | 1.97 | 1.59 | 1.66 | 2.07 | 1.66 | 1.82 | 1.66 |
| I felt as if the rubber hand was my hand | 1.33 | -1.37 | 0.59 | -1.19 | -1.74 | -2.15 | -1.52 | -2.11 | 1.63 | 1.87 | 1.71 | 2.01 | 1.74 | 1.24 | 1.71 | 1.48 |
| I felt as if my real hand were turning rubbery | -0.67 | -0.78 | -0.22 | -0.52 | -1.89 | -2.22 | -1.26 | -1.89 | 1.80 | 1.89 | 2.04 | 2.04 | 1.30 | 1.26 | 1.90 | 1.56 |
| It seems as if I had more than one right hand | -1.70 | -1.11 | -1.22 | -0.56 | -2.11 | -2.19 | -1.78 | -2.19 | 1.25 | 1.87 | 1.78 | 2.04 | 1.61 | 1.25 | 1.61 | 1.11 |
| It appeared as if the rubber hand were drifting  towards my real hand | -0.74 | -1.11 | -0.56 | -0.70 | -2.30 | -2.07 | -2.15 | -2.04 | 1.79 | 2.00 | 1.73 | 1.54 | 1.18 | 1.35 | 1.10 | 1.20 |
| It felt as if I had no longer a right hand, as if my  right hand had disappeared | -1.37 | -1.33 | -1.33 | -1.37 | -2.04 | -2.07 | -1.67 | -1.85 | 1.84 | 2.07 | 2.01 | 1.82 | 1.27 | 1.11 | 1.68 | 1.34 |
| The rubber hand moved just like I wanted it to,  as if it was obeying my will | 2.11 | -2.22 | -0.78 | -2.00 | 1.52 | -1.63 | -1.11 | -2.04 | 1.42 | 1.23 | 2.12 | 1.25 | 1.61 | 1.97 | 1.96 | 1.59 |
| I felt as if I was controlling the movements of  the rubber hand | 2.48 | -1.22 | -0.85 | -1.56 | 1.59 | -1.19 | -1.11 | -1.37 | 0.88 | 2.10 | 2.05 | 1.23 | 1.78 | 1.84 | 1.89 | 1.84 |
| I felt as if I was causing the movement I saw | 2.44 | -0.96 | -0.63 | -1.41 | 1.19 | -1.44 | -1.00 | -1.63 | 0.78 | 2.16 | 2.21 | 1.63 | 2.09 | 1.89 | 1.93 | 1.68 |
| Whenever I moved my finger I expected the  rubber finger to move in the same way | 2.52 | -0.48 | 0.89 | -1.07 | 2.15 | -0.15 | 0.33 | -0.85 | 0.78 | 2.32 | 2.18 | 1.55 | 1.14 | 2.47 | 2.11 | 1.96 |
| I felt as if the rubber hand was controlling my will | -1.96 | -1.04 | -0.07 | -0.67 | -2.48 | -1.52 | -0.81 | -1.22 | 1.59 | 2.14 | 2.19 | 2.13 | 0.93 | 1.76 | 2.15 | 1.80 |
| I felt as if the rubber hand was controlling my movements | -2.22 | -1.30 | 0.37 | -0.74 | -2.56 | -1.19 | -0.48 | -1.04 | 1.09 | 1.81 | 2.12 | 2.16 | 0.88 | 1.91 | 2.27 | 2.00 |
| I could sense the movement from somewhere  between my real hand and the rubber hand | -0.63 | -0.89 | -0.48 | -0.41 | -1.07 | -1.15 | -0.56 | -0.89 | 1.65 | 1.68 | 1.45 | 1.75 | 1.86 | 1.57 | 1.78 | 1.78 |
| It seemed as if the rubber hand had a will of its own | -2.30 | 1.11 | 0.26 | -0.04 | -2.04 | 0.89 | -0.30 | 1.04 | 1.07 | 2.06 | 2.16 | 2.24 | 1.39 | 2.02 | 2.38 | 2.00 |

Table 5-1. All peaks from the ownership contrast *[(P_M_S_T_C_O_–P_M_A_T_C_O_)–(P_M_S_T_I_O_–P_M_A_T_I_O_)] + [(A_M_S_T_C_O_–A_M_A_T_C_O_)–(A_M_S_T_I_O_–A_M_A_T_I_O_)]*. All peaks that survived the threshold p<0.005 (uncorrected, k≥10) are reported. Three peaks located in the white matter were excluded from this table.

| **cluster-level** | | | | **peak-level** | | | | | **MNI coordinates** |  |
| --- | --- | --- | --- | --- | --- | --- | --- | --- | --- | --- |
| p_FWE-corr_ | q_FDR-corr_ | K | p (uncorrected) | p_FWE-corr_ | q_FDR-corr_ | T | Z | p (uncorrected) | [X;Y;Z] | Anatomical localization |
| 0.11 | 0.25 | 479 | 0.01 | 0.24 | 0.99 | 5.01 | 4.20 | 0.000 | [-36;-32;66] | L Postcentral Gyrus |
|  |  |  |  | 0.43 | 0.99 | 4.66 | 3.98 | 0.000 | [-42;-10;58] | L Precentral Gyrus |
|  |  |  |  | 0.61 | 0.99 | 4.42 | 3.82 | 0.000 | [-34;-10;64] | L Precentral Gyrus |
| 0.97 | 0.92 | 95 | 0.18 | 0.28 | 0.99 | 4.91 | 4.14 | 0.000 | [-26;-76;42] | L IPS |
| 1.00 | 0.92 | 40 | 0.38 | 0.53 | 0.99 | 4.53 | 3.89 | 0.000 | [-16;-50;50] | L Precuneus? |
| 0.03 | 0.11 | 714 | 0.00 | 0.60 | 0.99 | 4.43 | 3.83 | 0.000 | [-24;42;38] | L dlPFC |
|  |  |  |  | 0.99 | 1.00 | 3.57 | 3.21 | 0.001 | [-16;54;28] | L Superior Frontal Gyrus |
|  |  |  |  | 1.00 | 1.00 | 3.52 | 3.18 | 0.001 | [-2;50;30] | L Superior Medial Gyrus |
| 0.79 | 0.92 | 166 | 0.08 | 0.84 | 1.00 | 4.11 | 3.60 | 0.000 | [4;-70;-12] | Cerebellum (Vermis 6) |
|  |  |  |  | 1.00 | 1.00 | 2.98 | 2.75 | 0.003 | [20;-64;-2] | R Lingual Gyrus |
|  |  |  |  | 1.00 | 1.00 | 2.96 | 2.74 | 0.003 | [26;-58;-4] | R Lingual Gyrus |
| 1.00 | 0.92 | 40 | 0.38 | 0.88 | 1.00 | 4.02 | 3.54 | 0.000 | [10;20;22] | R ACC |
| 0.97 | 0.92 | 89 | 0.19 | 0.94 | 1.00 | 3.87 | 3.44 | 0.000 | [-12;18;2] | L Caudate Nucleus |
| 1.00 | 0.92 | 56 | 0.30 | 0.98 | 1.00 | 3.73 | 3.33 | 0.000 | [12;48;-2] | R Mid Orbital Gyrus |
| 1.00 | 0.92 | 19 | 0.56 | 0.98 | 1.00 | 3.72 | 3.32 | 0.000 | [-48;-12;-26] | L Inferior Temporal Gyrus |
| 1.00 | 0.92 | 32 | 0.43 | 0.98 | 1.00 | 3.69 | 3.31 | 0.000 | [-60;-48;38] | L Inferior Parietal Lobule |
|  |  |  |  | 1.00 | 1.00 | 2.84 | 2.64 | 0.004 | [-54;-58;40] | L Inferior Parietal Lobule |
| 0.99 | 0.92 | 65 | 0.26 | 0.99 | 1.00 | 3.66 | 3.28 | 0.001 | [40;-74;-34] | R Cerebelum (VIIa) |
|  |  |  |  | 1.00 | 1.00 | 3.54 | 3.19 | 0.001 | [38;-72;-24] | R Cerebelum (VIIa) |
| 1.00 | 0.92 | 41 | 0.37 | 0.99 | 1.00 | 3.63 | 3.26 | 0.001 | [12;-44;38] | R Midcingulate cortex |
| 1.00 | 0.92 | 14 | 0.62 | 0.99 | 1.00 | 3.61 | 3.24 | 0.001 | [-10;-28;14] | Thalamus |
| 0.99 | 0.92 | 78 | 0.22 | 1.00 | 1.00 | 3.54 | 3.19 | 0.001 | [-24;-2;-4] | L Pallidum |
| 1.00 | 0.92 | 21 | 0.53 | 1.00 | 1.00 | 3.50 | 3.16 | 0.001 | [30;-10;-22] | R Hippocampus |
| 1.00 | 0.92 | 38 | 0.39 | 1.00 | 1.00 | 3.49 | 3.15 | 0.001 | [42;-68;-8] | R Inferior Temporal Gyrus |
| 1.00 | 0.92 | 48 | 0.34 | 1.00 | 1.00 | 3.49 | 3.15 | 0.001 | [-16;64;12] | L Superior Frontal Gyrus |
| 0.89 | 0.92 | 133 | 0.12 | 1.00 | 1.00 | 3.47 | 3.14 | 0.001 | [4;-68;-46] | R Cerebellum (VIIb) |
|  |  |  |  | 1.00 | 1.00 | 3.26 | 2.98 | 0.001 | [-2;-66;-52] | Cerebellum Lobule IX (Verm) |
|  |  |  |  | 1.00 | 1.00 | 3.09 | 2.84 | 0.002 | [18;-54;-52] | R Cerebelum (VIIIb) |
| 0.99 | 0.92 | 65 | 0.26 | 1.00 | 1.00 | 3.43 | 3.11 | 0.001 | [-50;24;0] | L Inferior Frontal Gyrus |
|  |  |  |  | 1.00 | 1.00 | 3.18 | 2.91 | 0.002 | [-54;28;10] | L Inferior Frontal Gyrus |
| 0.96 | 0.92 | 101 | 0.17 | 1.00 | 1.00 | 3.43 | 3.10 | 0.001 | [-4;50;8] | L Anterior Cingulate Cortex |
| 1.00 | 0.92 | 37 | 0.40 | 1.00 | 1.00 | 3.36 | 3.05 | 0.001 | [-10;38;-4] | L Anterior Cingulate Cortex |
|  |  |  |  | 1.00 | 1.00 | 3.10 | 2.85 | 0.002 | [-10;50;-4] | L Mid Orbital Gyrus |
| 0.98 | 0.92 | 79 | 0.22 | 1.00 | 1.00 | 3.35 | 3.05 | 0.001 | [26;-60;-20] | R Cerebelum (VI) |
|  |  |  |  | 1.00 | 1.00 | 3.31 | 3.01 | 0.001 | [18;-54;-18] | R Cerebelum (IV-V) |
| 1.00 | 0.92 | 57 | 0.29 | 1.00 | 1.00 | 3.35 | 3.04 | 0.001 | [26;-56;14] | Parieto-occipital fissure |
| 1.00 | 0.92 | 50 | 0.33 | 1.00 | 1.00 | 3.34 | 3.04 | 0.001 | [-6;-50;22] | L Posterior Cingulate Cortex |
| 1.00 | 0.92 | 19 | 0.56 | 1.00 | 1.00 | 3.34 | 3.04 | 0.001 | [32;-80;-42] | R Cerebelum (Crus 2) |
| 1.00 | 0.92 | 43 | 0.36 | 1.00 | 1.00 | 3.34 | 3.04 | 0.001 | [-54;0;-20] | L Middle Temporal Gyrus |
| 0.92 | 0.92 | 119 | 0.14 | 1.00 | 1.00 | 3.32 | 3.02 | 0.001 | [-18;-56;4] | L Calcarine Gyrus |
|  |  |  |  | 1.00 | 1.00 | 3.17 | 2.91 | 0.002 | [-12;-46;4] | L Calcarine Gyrus |
|  |  |  |  | 1.00 | 1.00 | 2.78 | 2.59 | 0.005 | [-6;-46;10] | L Cigulate Gyrus |
| 1.00 | 0.92 | 32 | 0.43 | 1.00 | 1.00 | 3.28 | 2.99 | 0.001 | [-38;48;-10] | L Middle Orbital Gyrus |
|  |  |  |  | 1.00 | 1.00 | 3.15 | 2.89 | 0.002 | [-38;40;-12] | L Inferior Frontal Gyrus |
| 1.00 | 0.92 | 26 | 0.48 | 1.00 | 1.00 | 3.24 | 2.96 | 0.002 | [16;-78;16] | R Calcarine Gyrus |
| 1.00 | 0.92 | 16 | 0.59 | 1.00 | 1.00 | 3.23 | 2.96 | 0.002 | [12;-52;-46] | R Cerebelum (IX) |
| 1.00 | 0.92 | 31 | 0.44 | 1.00 | 1.00 | 3.23 | 2.95 | 0.002 | [-60;-16;-20] | L Middle Temporal Gyrus |
| 1.00 | 0.92 | 15 | 0.60 | 1.00 | 1.00 | 3.23 | 2.95 | 0.002 | [-40;-56;-20] | L Fusiform Gyrus |
| 1.00 | 0.92 | 17 | 0.58 | 1.00 | 1.00 | 3.19 | 2.92 | 0.002 | [-44;18;46] | L Middle Frontal Gyrus |
| 1.00 | 0.92 | 13 | 0.63 | 1.00 | 1.00 | 3.18 | 2.91 | 0.002 | [-24;-78;-22] | L Cerebelum (Crus I) |
| 1.00 | 0.92 | 13 | 0.63 | 1.00 | 1.00 | 3.17 | 2.90 | 0.002 | [-38;18;20] | L Inferior Frontal Gyrus |
| 1.00 | 0.92 | 14 | 0.62 | 1.00 | 1.00 | 3.13 | 2.87 | 0.002 | [-46;-50;30] | L Angular Gyrus |
| 1.00 | 0.92 | 24 | 0.50 | 1.00 | 1.00 | 3.12 | 2.87 | 0.002 | [18;-80;4] | R Calcarine Gyrus |
| 1.00 | 0.92 | 11 | 0.66 | 1.00 | 1.00 | 3.02 | 2.79 | 0.003 | [24;-6;2] | R Pallidum |
| 1.00 | 0.92 | 30 | 0.45 | 1.00 | 1.00 | 3.01 | 2.77 | 0.003 | [12;24;-2] | R Caudate Nucleus |
|  |  |  |  | 1.00 | 1.00 | 2.85 | 2.65 | 0.004 | [18;16;-2] | R Putamen |
|  |  |  |  | 1.00 | 1.00 | 2.82 | 2.62 | 0.004 | [6;18;0] | R Caudate Nucleus |
| 1.00 | 0.92 | 11 | 0.66 | 1.00 | 1.00 | 2.97 | 2.74 | 0.003 | [44;-70;12] | R Middle Temporal Gyrus |

Table 7-1. All peaks from the agency contrast *[(A_M_S_T_C_O_–P_M_S_T_C_O_)–(A_M_A_T_C_O_–P_M_A_T_C_O_)] + [(A_M_S_T_I_O_–P_M_S_T_I_O_)–(A_M_A_T_I_O_–P_M_A_T_I_O_)].* All peaks that survived the threshold p<0.005 (uncorrected, k≥10) are reported.

| **cluster-level** | | | | **peak-level** | | | | | **MNI coordinates** |  |
| --- | --- | --- | --- | --- | --- | --- | --- | --- | --- | --- |
| p_FWE-corr_ | q_FDR-corr_ | K | p (uncorrected) | p_FWE-corr_ | q_FDR-corr_ | T | Z | p (uncorrected) | [X;Y;Z] | Anatomical localization |
| 0.05 | 0.09 | 790.00 | 0.00 | 0.01 | 0.03 | 6.44 | 5.00 | 0.00 | [-56;-26;16] | L Superior Temporal Gyrus |
|  |  |  |  | 0.04 | 0.06 | 5.75 | 4.63 | 0.00 | [-48;-30;12] | L Superior Temporal Gyrus |
|  |  |  |  | 0.69 | 0.57 | 4.17 | 3.65 | 0.00 | [-66;-26;4] | L Middle Temporal Gyrus |
| 0.41 | 0.51 | 347.00 | 0.04 | 0.15 | 0.15 | 5.12 | 4.27 | 0.00 | [60;-20;12] | R Superior Temporal Gyrus |
| 0.98 | 0.93 | 79.00 | 0.29 | 0.85 | 0.76 | 3.94 | 3.48 | 0.00 | [6;-40;50] | R Midcingulate Cortex |
| 1.00 | 0.93 | 12.00 | 0.70 | 0.88 | 0.76 | 3.89 | 3.45 | 0.00 | [-38;-8;62] | L Precentral Gyrus |
| 1.00 | 0.93 | 38.00 | 0.47 | 0.95 | 0.82 | 3.69 | 3.30 | 0.00 | [-36;-40;46] | L Inferior Parietal Lobule |
| 1.00 | 0.93 | 56.00 | 0.38 | 0.98 | 0.82 | 3.55 | 3.20 | 0.00 | [-26;-16;70] | L Precentral Gyrus |
|  |  |  |  | 1.00 | 0.84 | 3.08 | 2.84 | 0.00 | [-28;-24;70] | L Precentral Gyrus |
| 1.00 | 0.93 | 25.00 | 0.56 | 0.99 | 0.82 | 3.51 | 3.17 | 0.00 | [-52;-30;54] | L Postcentral Gyrus |
| 1.00 | 0.93 | 18.00 | 0.63 | 0.99 | 0.82 | 3.48 | 3.14 | 0.00 | [26;-58;62] | R Superior Parietal Lobule |
| 1.00 | 0.93 | 55.00 | 0.38 | 0.99 | 0.82 | 3.47 | 3.14 | 0.00 | [36;-40;52] | R IPS |
| 1.00 | 0.93 | 55.00 | 0.38 | 0.99 | 0.82 | 3.45 | 3.13 | 0.00 | [16;-42;-12] | R Lingual Gyrus |
| 1.00 | 0.93 | 32.00 | 0.51 | 0.99 | 0.82 | 3.42 | 3.10 | 0.00 | [12;-68;48] | R Precuneus |
| 1.00 | 0.93 | 12.00 | 0.70 | 1.00 | 0.82 | 3.34 | 3.04 | 0.00 | [-24;-42;60] | L Postcentral Gyrus |
| 1.00 | 0.93 | 47.00 | 0.42 | 1.00 | 0.82 | 3.34 | 3.04 | 0.00 | [6;-60;8] | R Lingual Gyrus |
| 1.00 | 0.93 | 22.00 | 0.59 | 1.00 | 0.84 | 3.26 | 2.97 | 0.00 | [-12;-42;46] | L Precuneus |
| 1.00 | 0.93 | 15.00 | 0.67 | 1.00 | 0.84 | 3.10 | 2.85 | 0.00 | [-36;-22;54] | L Central sulcus |
| 1.00 | 0.93 | 13.00 | 0.69 | 1.00 | 0.84 | 3.05 | 2.81 | 0.00 | [20;-44;-22] | R Cerebelum (V) |
| 1.00 | 0.93 | 17.00 | 0.64 | 1.00 | 0.84 | 3.05 | 2.81 | 0.00 | [-4;-56;58] | L Precuneus |

Table 9-1. All peaks from the ownership x agency interaction contrast *[(A_M_S_T_C_O_–P_M_S_T_C_O_)–(A_M_A_T_C_O_–P_M_A_T_C_O_)]–[(A_M_S_T_I_O_–P_M_S_T_I_O_)–(A_M_A_T_I_O_–P_M_A_T_I_O_)].* All peaks that survived the threshold p<0.005 (uncorrected, k≥10) are reported.

| **cluster-level** | | | | **peak-level** | | | | | **MNI coordinates** |  |
| --- | --- | --- | --- | --- | --- | --- | --- | --- | --- | --- |
| p_FWE-corr_ | q_FDR-corr_ | K | p (uncorrected) | p_FWE-corr_ | q_FDR-corr_ | T | Z | p (uncorrected) | [X;Y;Z] | Anatomical localization |
| 0.76 | 0.93 | 194.00 | 0.09 | 0.70 | 0.99 | 4.21 | 3.67 | 0.00 | [-38;-28;52] | L Postcentral Gyrus |
|  |  |  |  | 1.00 | 0.99 | 3.11 | 2.86 | 0.00 | [-36;-14;50] | L Precentral Gyrus |
|  |  |  |  | 1.00 | 0.99 | 2.94 | 2.72 | 0.00 | [-44;-20;46] | L Postcentral Gyrus |
| 1.00 | 0.93 | 14.00 | 0.66 | 1.00 | 0.99 | 3.42 | 3.10 | 0.00 | [-26;-30;70] | L Postcentral Gyrus |
| 0.97 | 0.93 | 98.00 | 0.22 | 1.00 | 0.99 | 3.40 | 3.09 | 0.00 | [-60;-16;12] | L Superior Temporal Gyrus |
| 1.00 | 0.93 | 21.00 | 0.58 | 1.00 | 0.99 | 3.39 | 3.08 | 0.00 | [20;-44;-12] | R Fusiform Gyrus |
| 1.00 | 0.93 | 10.00 | 0.72 | 1.00 | 0.99 | 3.08 | 2.83 | 0.00 | [-54;-30;50] | L Inferior Parietal Lobule |
| 1.00 | 0.93 | 11.00 | 0.70 | 1.00 | 0.99 | 3.00 | 2.77 | 0.00 | [46;-18;16] | R Parietal Operculum |
| 1.00 | 0.93 | 10.00 | 0.72 | 1.00 | 0.99 | 2.94 | 2.73 | 0.00 | [-38;-18;2] | L Insula |

Table 10-1. All peaks from the inverse of the ownership x agency interaction contrast *[(A_M_S_T_C_O_–P_M_S_T_C_O_)–(A_M_A_T_C_O_–P_M_A_T_C_O_)]–[(A_M_S_T_I_O_–P_M_S_T_I_O_)–(A_M_A_T_I_O_–P_M_A_T_I_O_)].* All peaks that survived the threshold p<0.005 (uncorrected, k≥10) are reported.

| **cluster-level** | | | | **peak-level** | | | | | **MNI coordinates** |  |
| --- | --- | --- | --- | --- | --- | --- | --- | --- | --- | --- |
| p_FWE-corr_ | q_FDR-corr_ | K | p (uncorrected) | p_FWE-corr_ | q_FDR-corr_ | T | Z | p (uncorrected) | [X;Y;Z] | Anatomical localization |
| 0.95 | 0.93 | 114.00 | 0.19 | 0.79 | 0.58 | 4.08 | 3.58 | 0.00 | [-14;-98;2] | L Middle Occipital Gyrus |
|  |  |  |  | 0.94 | 0.58 | 3.77 | 3.36 | 0.00 | [-22;-92;-2] | L Middle Occipital Gyrus |
|  |  |  |  | 1.00 | 0.74 | 3.14 | 2.88 | 0.00 | [-22;-86;8] | Transverse Occipital Sulcus |
| 1.00 | 0.93 | 13.00 | 0.67 | 1.00 | 0.74 | 3.20 | 2.93 | 0.00 | [-34;-80;-2] | L Middle Occipital Gyrus |
| 1.00 | 0.93 | 18.00 | 0.61 | 1.00 | 0.74 | 3.08 | 2.84 | 0.00 | [26;-92;4] | R Middle Occipital Gyrus |

Table 12-1. The peaks from the localizer experiment used to define the ROIs in the current study.

| **Peaks from localizer experiment** | | | | |
| --- | --- | --- | --- | --- |
| Anatomical region | MNI coordinates | | | Peak t |
|  | **x** | **y** | **z** |  |
| L Postcentral gyrus | -38 | -20 | 52 | 8.41 |
| L Precentral gyrus | -44 | -10 | 56 | 7.46 |
| L Precentral gyrus | 46 | -12 | 56 | 4.55 |
| L inferior parietal lobule | -56 | -52 | 44 | 5.18 |
| R Superior temporal gyrus | 48 | -40 | 16 | 3.88 |
| R Middle temporal gyrus | 50 | -70 | -2 | 9.25 |
| R Cerebellum | 16 | -54 | -24 | 5.90 |
| R Cerebellum | 38 | -70 | -28 | 4.55 |
| R Cerebellum | 14 | -68 | -46 | 4.40 |

Table 13-1 . Frequency of taps per condition and pre-illusion period.

| **Frequency of taps per condition (period used in fMRI analysis) and pre-illusion period (Hz)** | | | | | | | | | | | | | | | | |
| --- | --- | --- | --- | --- | --- | --- | --- | --- | --- | --- | --- | --- | --- | --- | --- | --- |
| Participant no | Pre- | Active/ Synchronous/ Congruent | Pre- | Active/ Asychronous/ Congruent | Pre- | Passive/ Synchronous/ Congruent | Pre- | Passive/ Asynchronous/ Congruent | Pre- | Active/ Synchronous/ Incongruent | Pre- | Active/ Asynchronous/ Incongruent | Pre- | Passive/ Synchronous/ Incongruent | Pre- | Passive/ Asynchronous/ Incongruent |
| 10 | 1.81 | 1.37 | 1.96 | 1.56 | 1.54 | 1.36 | 1.67 | 1.37 | 1.98 | 1.63 | 2.36 | 1.49 | 1.49 | 1.40 | 1.87 | 1.61 |
| 11 | 0.58 | 0.79 | 0.31 | 0.40 | 0.57 | 0.82 | 0.47 | 0.47 | 0.39 | 0.36 | 0.40 | 0.18 | 0.34 | 0.25 | 0.42 | 0.27 |
| 12 | 0.81 | 0.73 | 1.26 | 1.20 | 1.14 | 1.05 | 0.82 | 0.78 | 0.96 | 0.89 | 1.81 | 1.39 | 1.26 | 1.16 | 0.93 | 0.88 |
| 13 | 0.00 | 1.70 | 0.00 | 1.79 | 0.00 | 1.71 | 0.00 | 1.97 | 0.00 | 1.43 | 0.00 | 1.66 | 0.00 | 1.53 | 0.00 | 1.51 |
| 14 | 1.41 | 1.48 | 1.30 | 1.66 | 1.57 | 1.57 | 1.60 | 1.64 | 1.79 | 1.66 | 2.16 | 1.65 | 1.66 | 1.56 | 1.77 | 1.67 |
| 15 | 1.31 | 0.98 | 1.52 | 1.35 | 1.82 | 1.62 | 1.42 | 1.15 | 1.72 | 1.33 | 2.27 | 1.68 | 1.80 | 1.44 | 1.77 | 1.37 |
| 16 | 1.46 | 1.25 | 1.68 | 1.43 | 1.81 | 1.73 | 1.75 | 1.52 | 1.80 | 1.54 | 1.76 | 1.18 | 1.15 | 1.02 | 1.62 | 1.38 |
| 17 | 1.60 | 1.48 | 1.91 | 1.71 | 1.72 | 1.65 | 1.52 | 1.38 | 1.70 | 1.62 | 2.39 | 1.82 | 1.80 | 1.98 | 1.66 | 1.57 |
| 18 | 1.28 | 1.41 | 1.51 | 1.70 | 1.75 | 1.63 | 1.62 | 1.67 | 1.82 | 1.62 | 3.04 | 2.02 | 2.10 | 1.72 | 2.01 | 1.66 |
| 19 | 2.00 | 1.89 | 2.06 | 1.93 | 1.88 | 1.83 | 1.96 | 1.74 | 2.30 | 2.24 | 2.34 | 1.65 | 1.00 | 0.95 | 2.01 | 1.98 |
| 20 | 0.00 | 1.25 | 0.00 | 1.45 | 0.00 | 1.74 | 0.00 | 1.41 | 0.00 | 1.15 | 0.00 | 1.55 | 0.00 | 1.40 | 0.00 | 1.27 |
| 21 | 1.04 | 1.29 | 0.67 | 0.63 | 0.52 | 0.49 | 1.54 | 1.41 | 1.71 | 1.78 | 2.57 | 2.10 | 1.79 | 1.66 | 1.88 | 1.70 |
| 22 | 1.65 | 1.45 | 2.02 | 1.80 | 1.97 | 1.75 | 1.66 | 1.55 | 1.89 | 1.70 | 2.79 | 2.04 | 2.09 | 1.96 | 1.84 | 1.76 |
| 23 | 2.15 | 1.77 | 2.26 | 2.13 | 1.24 | 0.77 | 2.42 | 1.97 | 1.98 | 1.85 | 2.58 | 2.08 | 2.03 | 1.94 | 2.19 | 2.00 |
| 24 | 1.79 | 1.58 | 2.05 | 1.84 | 1.84 | 1.51 | 1.84 | 1.61 | 1.19 | 1.46 | 2.11 | 1.70 | 2.00 | 1.72 | 1.69 | 1.61 |
| 25 | 2.02 | 1.70 | 2.11 | 1.63 | 2.09 | 1.73 | 2.06 | 1.65 | 2.02 | 1.73 | 2.25 | 1.77 | 1.92 | 1.45 | 2.01 | 1.73 |
| 26 | 2.13 | 1.92 | 2.14 | 1.97 | 2.11 | 1.92 | 2.12 | 1.88 | 1.95 | 1.70 | 2.59 | 1.69 | 2.00 | 1.82 | 2.00 | 1.74 |
| 27 | 1.83 | 1.35 | 1.91 | 1.67 | 1.75 | 1.45 | 2.24 | 1.69 | 1.33 | 1.98 | 2.14 | 2.10 | 1.41 | 1.85 | 1.37 | 2.04 |
| 28 | 0.69 | 1.22 | 1.07 | 1.41 | 1.45 | 1.64 | 1.07 | 1.34 | 1.04 | 1.41 | 1.61 | 1.57 | 1.60 | 1.60 | 1.46 | 1.60 |
| 29 | 1.94 | 1.80 | 2.21 | 1.94 | 2.04 | 1.82 | 2.02 | 1.48 | 3.10 | 2.81 | 3.02 | 1.87 | 2.58 | 2.21 | 2.27 | 1.92 |
